# Age, Spatial, and Temporal Variations in Hospital Admissions with Malaria in Kilifi County, Kenya: A 25-Year Longitudinal Observational Study

**DOI:** 10.1371/journal.pmed.1002047

**Published:** 2016-06-28

**Authors:** Polycarp Mogeni, Thomas N. Williams, Gregory Fegan, Christopher Nyundo, Evasius Bauni, Kennedy Mwai, Irene Omedo, Patricia Njuguna, Charles R. Newton, Faith Osier, James A. Berkley, Laura L. Hammitt, Brett Lowe, Gabriel Mwambingu, Ken Awuondo, Neema Mturi, Norbert Peshu, Robert W. Snow, Abdisalan Noor, Kevin Marsh, Philip Bejon

**Affiliations:** 1 KEMRI-Wellcome Trust Research Programme, Kilifi, Kenya; 2 Imperial College London, London, United Kingdom; 3 Centre for Tropical Medicine and Global Health, Nuffield Department of Clinical Medicine, University of Oxford, CCVTM, Oxford, United Kingdom; 4 Johns Hopkins Bloomberg School of Public Health, Department of International Health, Baltimore, Maryland, United States of America; 5 Spatial Health Metrics Group, Kenya Medical Research Institute/Wellcome Trust Research Programme, Nairobi, Kenya; Epicentre, FRANCE

## Abstract

**Background:**

Encouraging progress has been seen with reductions in *Plasmodium falciparum* malaria transmission in some parts of Africa. Reduced transmission might lead to increasing susceptibility to malaria among older children due to lower acquired immunity, and this has implications for ongoing control strategies.

**Methods and Findings:**

We conducted a longitudinal observational study of children admitted to Kilifi County Hospital in Kenya and linked it to data on residence and insecticide-treated net (ITN) use. This included data from 69,104 children aged from 3 mo to 13 y admitted to Kilifi County Hospital between 1 January 1990 and 31 December 2014. The variation in malaria slide positivity among admissions was examined in logistic regression models using the following predictors: location of the residence, calendar time, the child’s age, ITN use, and the enhanced vegetation index (a proxy for soil moisture). The proportion of malaria slide-positive admissions declined from 0.56 (95% confidence interval [CI] 0.54–0.58) in 1998 to 0.07 (95% CI 0.06–0.08) in 2009 but then increased again through to 0.24 (95% CI 0.22–0.25) in 2014. Older children accounted for most of the increase after 2009 (0.035 [95% CI 0.030–0.040] among young children compared to 0.22 [95% CI 0.21–0.23] in older children). There was a nonlinear relationship between malaria risk and prevalence of ITN use within a 2 km radius of an admitted child’s residence such that the predicted malaria positive fraction varied from ~0.4 to <0.1 as the prevalence of ITN use varied from 20% to 80%. In this observational analysis, we were unable to determine the cause of the decline in malaria between 1998 and 2009, which pre-dated the dramatic scale-up in ITN distribution and use.

**Conclusion:**

Following a period of reduced transmission, a cohort of older children emerged who have increased susceptibility to malaria. Further reductions in malaria transmission are needed to mitigate the increasing burden among older children, and universal ITN coverage is a promising strategy to achieve this goal.

## Abstract 2

Using observational data over 25 years, Polycarp Mogeni and colleagues examine the age, spatial, and temporal variations in malaria hospital admissions in Kilifi County, Kenya.

## Author Summary

**Why Was This Study Done?:**

- The past decade has seen a marked decline in malaria transmission in some parts of Africa alongside substantial investments to control malaria. However, malaria remains a public health emergency in sub-Saharan Africa.
- As malaria declines in some parts of Africa, older children may become more susceptible to clinical malaria because reduced exposure earlier in life decreases opportunities for acquired immunity.
- Insecticide-treated net (ITN) use in randomized controlled trials and observational studies of effectiveness has been associated with reduced malaria morbidity and mortality.
- Given the ongoing global campaign to increase coverage, there is need for continuous assessment of ITN effectiveness in field conditions.

**What Did the Researchers Do and Find?:**

- We analyzed 25 y of longitudinal surveillance data of hospital admissions to Kilifi County Hospital in the Kenyan Coast between 1990 and 2014. We included 69,104 children between 3 mo and 13 y of age in the analysis.
- We observed a decline in the proportion of admitted children with malaria parasites on blood testing from 1998 to 2009. However, there was a steady and marked increase in the proportion of admitted children with malaria parasites in their blood from 2009 to 2014. This coincided with a shift of malaria burden from younger to older children. Fortunately, there was no increase in malaria-related mortality.
- The proportion of children with malaria parasites was consistently higher in the southern region of Kilifi County compared to the north, thereby providing an opportunity for targeted interventions. However, within the northern and southern regions there was substantial geographical variability from year to year, which would complicate targeted control.
- Community ITN use around the admitted child’s residence was highly effective—i.e., children presenting for admission surrounded by high ITN usage at the community level were less likely to present with malaria parasites compared with those surrounded by low ITN usage.

**What Do These Findings Mean?:**

- These findings suggest that further reductions of malaria transmission are needed to mitigate the increasing burden among older children and that universal ITN coverage is a promising strategy to achieve this goal.

## Introduction

Some parts of Africa have seen marked reductions in malaria transmission [1–3] with associated reductions in malaria-related morbidity and mortality. Children acquire immunity to malaria following repeated infections, and hence, there is an inverse relationship between the intensity of malaria transmission and age of susceptibility to malaria [4]. Reduced transmission intensity of infectious diseases has been associated with increasing age of susceptibility [5]. This trend has been reported in malaria in both field conditions [6–8] and simulation studies incorporating acquired immunity [9]. For instance, reductions in malaria transmission led to more marked improvements in outcomes for children under 5 y than in the older age groups in Rwanda [7]. In Senegal, a rebound in malaria attacks was observed among adults and older children following a period of declining transmission [8]. These observations may be attributed to reduced population-levels of immunity due to reduced exposure. It is therefore essential to monitor outcomes following initial reductions in malaria transmission.

The objective of the analysis was to describe trends of malaria admissions by age group and examine the efficacy of community insecticide-treated net (ITN) use. We report here an analysis of 69,104 hospital admissions over 25 y from 1990 to 2014. Data for the last 6 y were linked to detailed data on ITN use collected through a demographic surveillance of ~260,000 residents. We also examined the enhanced vegetation index (EVI) as a proxy for soil moisture content, which may correlate with the presence of mosquito breeding sites [10].

## Methods

Approval for human participation in the hospital surveillance and the cohorts was given by the Kenya Medical Research Institute (KEMRI) Scientific Steering Committee and the Ethical Review Committee of KEMRI. Written informed consent was obtained from the parents/guardians of the children admitted to hospital. If participants could not write, a thumb-print was obtained, with a witness countersigning that the contents of the information sheet had been explained. The studies were conducted according to the principles of the Declaration of Helsinki.

### Study Area

The study was conducted at Kilifi County Hospital, which is situated at the center of the Kilifi Health and Demographic Surveillance System (KHDSS) area on the Kenyan Coast. The KHDSS covers a population of ~260,000 people living in an area of ~891 km^2^, as described previously [11]. Screening for malaria parasites has been continuous for 25 y since the establishment of a pediatric admission ward surveillance system in 1989.

### Data Collection

Children below 13 y of age were admitted to the pediatric service, where demographic details and clinical history were recorded and blood was examined for malaria parasites by microscopy. These assessments were conducted on all emergency admissions, irrespective of presumptive diagnosis, with the exception of those admitted for elective surgery.

Children with signs of severe disease such as impaired consciousness or deep breathing were admitted to the high-dependency ward, and children without such signs were admitted to the pediatric ward. Research clinicians provided 24-h clinical cover of both the high-dependency unit and the general pediatric ward.

Thick and thin blood smears were stained with 10% Giemsa and examined at 1,000x magnification for malaria parasites. One hundred microscopy fields were examined before slides could be considered negative. Microscopy standards were monitored through a quality assurance scheme. The scheme included a comprehensive microscopy training done as part of the induction and at regular intervals during the study period. The training also included the use of external quality control slides.

### Data Analysis

Our prespecified protocol was to describe the variation in malaria positive fraction in space and time within Kilifi County. Here, we present a data-driven analysis of the trends observed towards increasing malaria in older children over time. The analysis included children aged from 3 mo to 13 y who were admitted to Kilifi County Hospital between 1990 and 2014 and were residents of the KHDSS area. Key metrics were the malaria positive fraction (MPF) (i.e., the fraction of slide positives among all admitted children) and the mean age of the children admitted with positive malaria slides (i.e., without applying a threshold parasitemia). Thus, “Malaria” included all acute admissions with positive slides. We examined the correlation between the MPFs from passive surveillance at the hospital with incidence data from active surveillance of two cohorts in the community, conducted as previously described [12,13]. Unpaired *t*-test or the Wilcoxon rank-sum test was used where appropriate to compare average/median age between groups. Multiple fractional polynomials were used to assess nonlinear associations in regression models. A sensitivity analysis was carried out with “malaria case” defined using a cut-off parasitemia of >2,500 parasites per μl.

### Spatiotemporal Models

The spatiotemporal heterogeneity of malaria transmission in Kilifi County was assessed using a multivariable logistic regression model with the presence or absence of malaria parasites by microscopy among medical admissions (excluding trauma and elective surgery) as the outcome variable and time (continuous variable to capture the secular trends), year (categorical to capture year-to-year variability), region (referring to north versus south of the creek), location (referring to administrative areas within region), and space–time interactions as the independent covariates. We used the Pseudo R^2^ to assess the contribution of the various components of the model. As a measure for the background community prevalence of parasitemia, age-standardized parasite prevalence estimates among trauma cases were determined using standard methods [14], but only when children were afebrile on admission and clinical assessment did not reveal any other acute cause for admission.

### Covariate Data

EVI data available between 2000 and 2014 were used as a proxy for soil moisture [10]. They were derived from the National Aeronautics and Space Administration’s (NASA’s) remote-sensing Moderate-Resolution Imaging Spectroradiometer (MODIS) instrument on board the Terra satellites [15]. EVI data were downloaded at a pixel resolution of 250 x 250 m^2^ for 16-d intervals and then averaged by year. Data on ITN use were obtained from yearly KHDSS surveys of all residents conducted from 2009 through 2014. We used data from responses to the question “Did you sleep under an ITN last night?” or “Did your child sleep under an ITN last night?”

We imposed regular geographical grids on the study area and concentric circles around each admitted child’s residence using longitude and latitude coordinates. The prevalence of ITN use within each concentric circle and the mean EVI within each grid space were used in multivariable models. We selected a parsimonious model from the set of EVI and ITN covariates created through a stepwise model selection criterion. We tested for the superiority of nonlinear models over linear models by plotting residuals against covariates and testing the significance of multiple fractional polynomials over linear fits where asymmetrical distributions of residuals were identified. Observations with missing locations, ages, and slide results were excluded prior to the analysis. We used multiple imputation with chained equations in STATA software with 50 imputations to impute missing data on personal ITN use. To impute personal ITN use, the outcome variable (malaria slide result) and the covariates (EVI, age, and community ITN use) were included in the multiple imputation model. Multiple fractional polynomial transformation was applied to allow for nonlinearity in continuous variable effects (EVI, age, and community ITN use) in the imputation model. The multivariable logistic regression model results for the imputed datasets (combined using Rubin’s rules) were then compared to model results from the complete case analysis. Age-standardized parasite prevalence was calculated in R (version 3.0.2) [16], and all other analyses were done using Stata 12.0 (Stata, College Station, Texas, United States).

## Results

### Geographical Distribution and Trends in MPF

**Fig 1:**
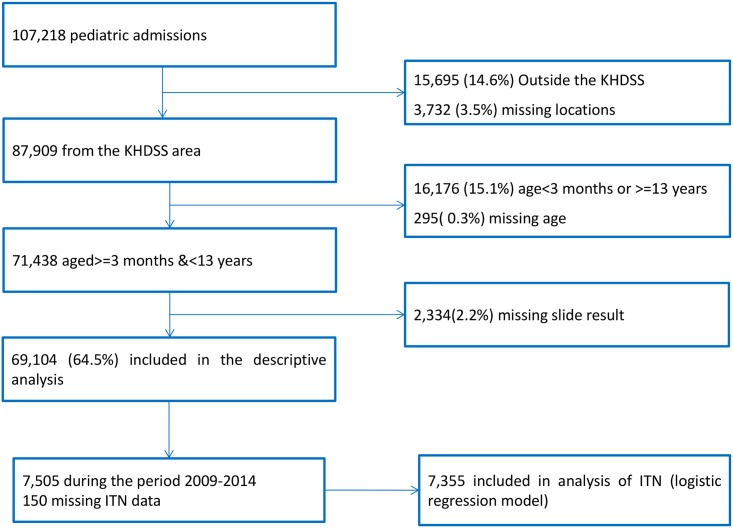
Flow diagram of participant numbers. Participant numbers and reasons for exclusion at each stage are shown.

**Fig 2:**
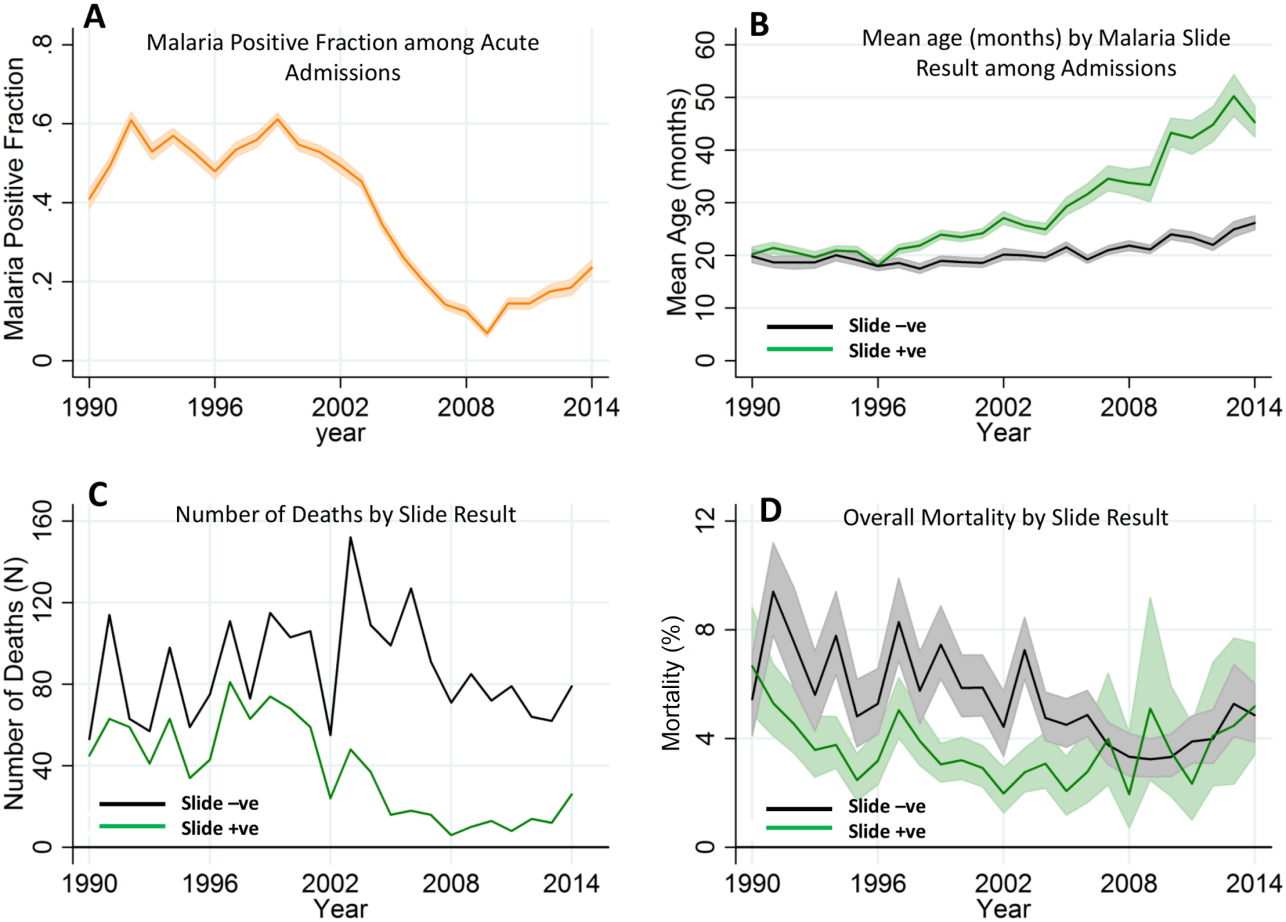
Temporal trends of malaria positive fraction (MPF), age of slide positivity, and mortality among acute admissions. Panel A shows the temporal trend of MPF. Panel B shows the trends of mean age over time for the slide-positive and slide-negative admissions. Panel C shows the temporal trends of absolute number of deaths, and Panel D shows case fatality rates. Shaded areas in panels A, B, and D represent 95% CIs.

Over 25 y, there were 107,218 pediatric admissions recorded, of whom 87,909 were known residents of the KHDSS and 71,438 were ≥3 mo or <13 y of age. Malaria slide data were missing for 2,334, leaving 69,104 for our analysis (Fig 1). The characteristics of the study population are presented in Table 1. The fraction of acute admissions that were positive for malaria (i.e., the MPF) for the full dataset was 0.389 (95% CI 0.385–0.393). MPF was subject to marked spatial and temporal heterogeneity (S1 Video and Fig 2A).

**Table 1:** Characteristics of the study population.

Period in Which Data Were Available	Analysis Type	Characteristics	Malaria Slide Positive	Malaria Slide Negative
1990 to 2014	Descriptive Analysis	Admissions *n* (%)	26,883 (38.9)	42,221 (61.1)
		Age median (IQR)	25.9 (13.2–44.9)	19.2 (9.7–42.7)
		Male, *n* (%)	14,217 (53.0)	23,862 (56.6)
		**Locations**		
		Chonyi *n* (%)	2,774 (10.3)	3,955 (9.4)
		Gede *n* (%)	695 (2.6)	1,856 (4.4)
		Jaribuni *n* (%)	2,018 (7.5)	2,383 (5.7)
		Junju *n* (%)	2,018 (7.5)	3,038 (7.2)
		Mtwapa *n* (%)	1,182 (4.4)	3,160 (7.5)
		Ngerenya *n* (%)	1,246 (4.6)	1,941 (4.6)
		Roka *n* (%)	1,964 (7.3)	2,960 (7.0)
		Sokoke *n* (%)	1,470 (5.5)	1,861 (4.4)
		Takaungu *n* (%)	5,071 (18.9)	4,590 (10.9)
		Tezo *n* (%)	8,404 (31.3)	16,437 (39.0)
2009 to 2014	Logistic Regression Model	Admissions *n* (%)	1,294 (17.2)	6,211(82.8)
		Age Median (IQR)	48.1 (30.1–73.1)	24.2 (11.8–54.3)
		Missing Personal ITN Use *n* (%)	47(3.6)	103(1.7)
		Missing Data on ITN Use during Community Surveys *n* (%)	190,417 (12.8%)	

IQR, interquartile range.

The average age of children admitted with malaria-positive slides and acute illness increased gradually from 20.2 mo (95% CI 18.9–21.6) in 1990 to 45.3 mo (95% CI 42.5–48.3) in 2014 (*p* < 0.001). During the same period, the mean age for children with malaria-negative slides and acute illness increased only slightly from 19.8 mo (95% CI 18.6–21.1) in 1990 to 26.1 mo (95% CI 24.9–27.5, *p* < 0.001) in 2014 (Fig 2B). Furthermore, the mean age of children with asymptomatic parasitemia among trauma admissions did not increase significantly, ranging from a geometric mean of 52.1 mo (95% CI 49.5–54.9) in the 1990–2002 predecline period to 64.6 mo of age (95% CI 49.5–84.2) in the 2009–2014 postdecline period (*p* = 0.0543; the latter wide CI reflects the reduced prevalence of asymptomatic infection).

The absolute number of malaria slide-positive deaths recorded in the hospital surveillance fell over time, although the absolute number of malaria slide-negative deaths did not show a clear trend (Fig 2C). The case fatality rate (i.e., the proportion of children admitted who died) among malaria slide-positive children was static, although among slide negative-children, the case fatality rate showed a gradual decline (Fig 2D).

**Fig 3:**
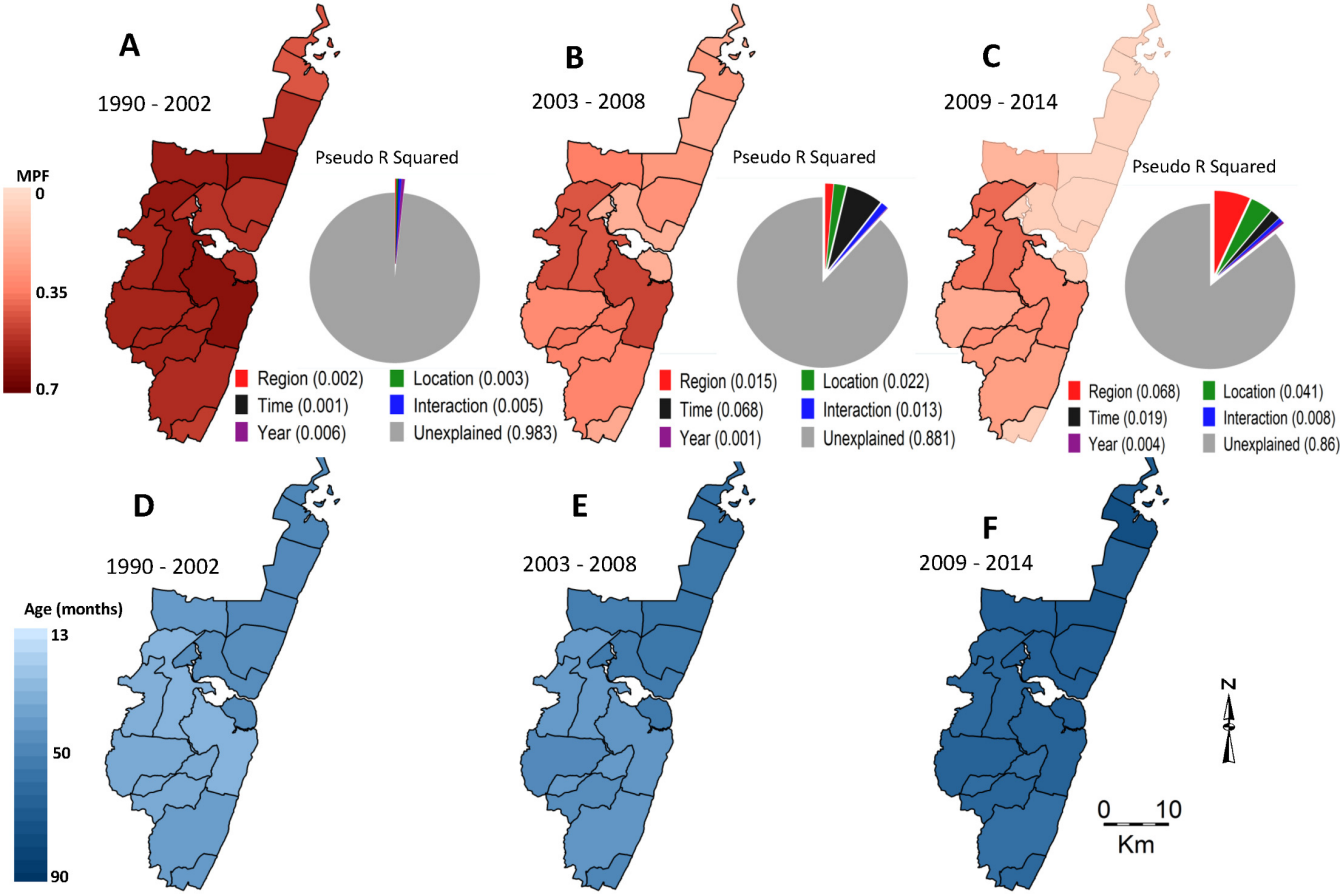
Geographical distribution of MPF and age of slide-positive acute admissions over time. Panels A, B, and C show spatial distributions of MPF for the predecline, decline, and postdecline periods respectively, and their associated pie charts show the proportion of the variability in MPF explained by the predictors: region (i.e., north versus south), location, time trend (i.e., as a continuous variable), an interaction between time and location, year to year variation (i.e., year as a stratified variable), and the unexplained variations. Panels D, E, and F show spatial distributions of age in months for the slide-positive admissions during the predecline, decline, and postdecline periods.

There was a decline in MPF over the years, with the lowest MPF (i.e., 0.07 [198/2,858]) being recorded in 2009, and a subsequent increase in MPF (up to 0.24 [511/2,169]) in 2014 (Fig 2A). We therefore refer to the 1990–2002, 2003–2008, and 2009–2014 as “predecline,” “decline,” and “postdecline” periods, respectively. Models accounted for variation in MPF poorly in the predecline period; however, the decline period was dominated by temporal variation, and the postdecline period was dominated by spatial variation (Fig 3A, 3B and 3C, respectively).

There was an inverse relationship between MPF and the average age of children with positive slides when averaged by location: areas with a higher average age of malaria tended to have lower MPF and vice versa (Fig 3A compared to 3D, 3B compared to 3E, and 3C compared to 3E). This was significant before the decline (r = −0.36, *p* < 0.001), but not after (r = −0.05, *p* = 0.66). There was a shift in the burden of disease from younger age groups to older age groups after the decline (S1 Fig).

### Access to Care

In order to assess the effect of access to care, we examined the data for trends in MPF over the euclidean distance between children’s residence and the hospital (S2 Fig). Best-fit lines showed greater heterogeneity in MPF over distance than linear trend in MPF over distance (a nonsignificant gradient of –0.003 per km, [95% CI −0.006–0.0003, *p* = 0.08]), suggesting that geographical heterogeneity had a greater effect than any bias that may have been introduced by distance from the hospital. Furthermore, we found that the incidence of malaria on active case detection from two cohorts monitored over 10 y [12,13] correlated closely with MPF measured at the hospital (r = 0.84, *p* < 0.001, S3 Fig).

### Is the Postdecline Increase in Malaria Due to Increasing Transmission or Falling Immunity?

**Fig 4:**
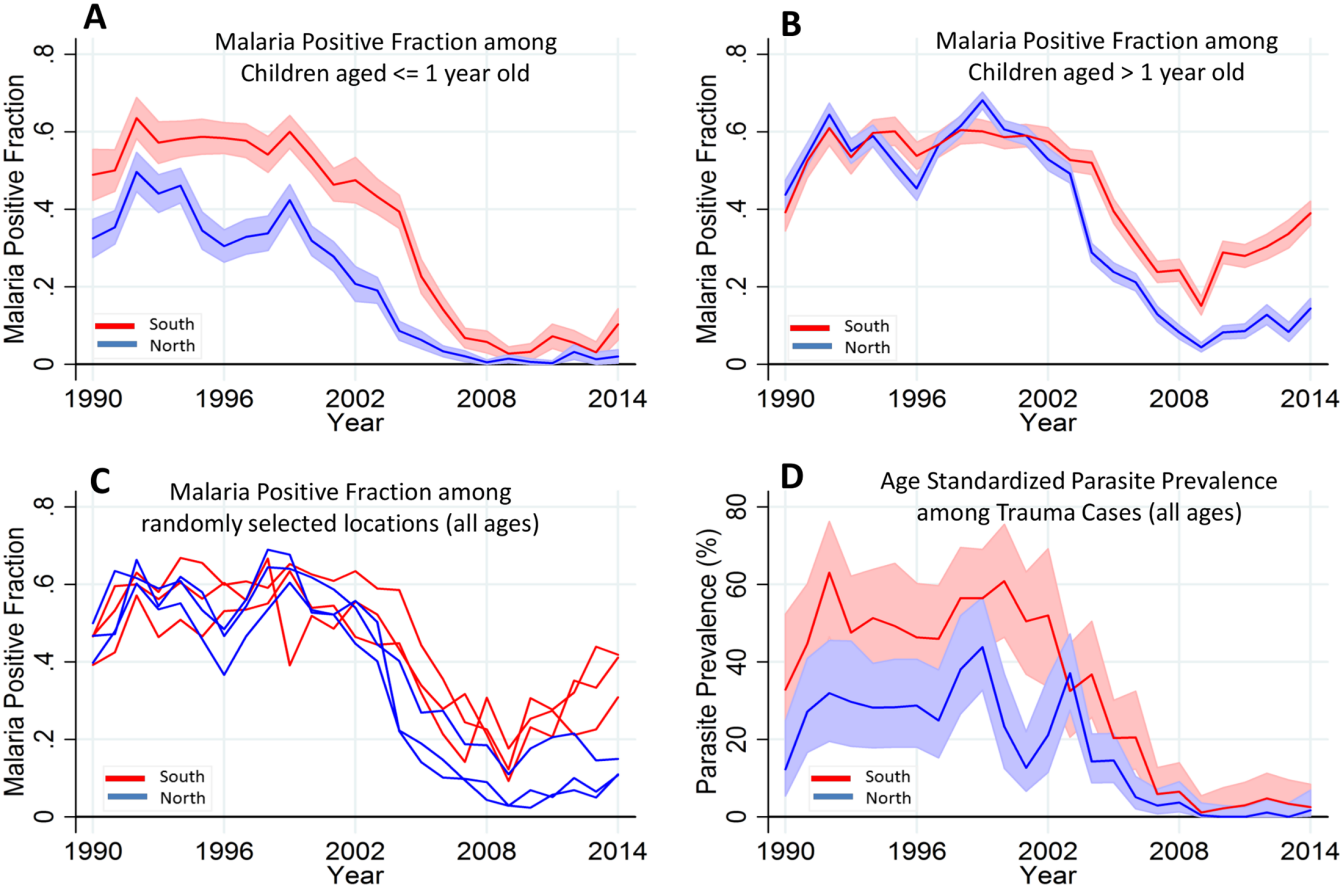
Temporal trends of MPF by age and parasite prevalence. Panels A and B show the temporal trends of MPF in admitted children aged ≤ 1 y old and children aged > 1 y old, respectively; the red line represents the southern region, while the blue line represents the northern region of the creek. Panel C shows all age MPF for randomly selected locations from the southern and northern regions. Panel D shows the age-standardized parasite prevalence (i.e., *Plasmodium falciparum* parasite rate [PfPR_2-10_]) among the trauma cases. Shaded areas in panels A, B, and D represent 95% CIs.

In order to disentangle the effects of loss of immunity from transmission intensity, we hypothesized that MPF for children ≤1 y old (MPF_<1yr_) would indicate transmission intensity without the offsetting of acquired immunity (as has been done previously [17,18]). We examined the trends in MPF_<1yr_ by region (i.e., using the creek in the center of the county as the point of division into northern versus southern regions) in view of the significant effect of region on variability of MPF (Fig 3C). MPF_<1yr_ showed a clear decline from the mid-1990s, down to almost zero in the northern region by 2008 and to low levels in the southern region from 2008 onwards, with a very slight increase after 2009 in the North and a slightly more marked but variable increase in the South after 2009 (Fig 4A). By comparison, MPF_>1yr_ showed a greater increase in both regions in the postdecline period, which was particularly marked in the South (Fig 4B). Within geographical locations, malaria positivity showed substantial variability from year to year (Fig 4C).

We also examined the age-corrected parasite prevalence (PfPR_2-10_) among the 5.8% (3,971/69,104) that were trauma admissions (excluding trauma cases with fever) as a proxy of community parasite prevalence (Fig 4D). PfPR_2-10_ declined from 43% (30/72) and 56% (35/62) in the northern and southern regions, respectively, in 1999, to <1% (i.e., 0/151 in the North and 1/100 in the South) in both regions in 2009 but subsequently rose, reaching 2% (2/102) and 4% (3/83) in the northern and southern regions, respectively, in 2014.

### Factors Predicting Malaria-Positive Slides among Admissions

Over the full monitoring period from 1990–2014, there was significant variation in MPF over space and time, and the spatial variation was most marked in the postdecline period (Fig 3C). The variation accounted for by an interaction between space and time was also more marked during this period, suggesting substantial temporal instability of the subregional spatial pattern during this period (Fig 4D). The interaction between space and time was strongly significant (*p* < 0.001) and accounted for ~1% of the variability in MPF. However, the regional differences (i.e., with lower MPFs in the north versus the south of the creek) remained temporally consistent despite the subregional instability.

**Fig 5:**
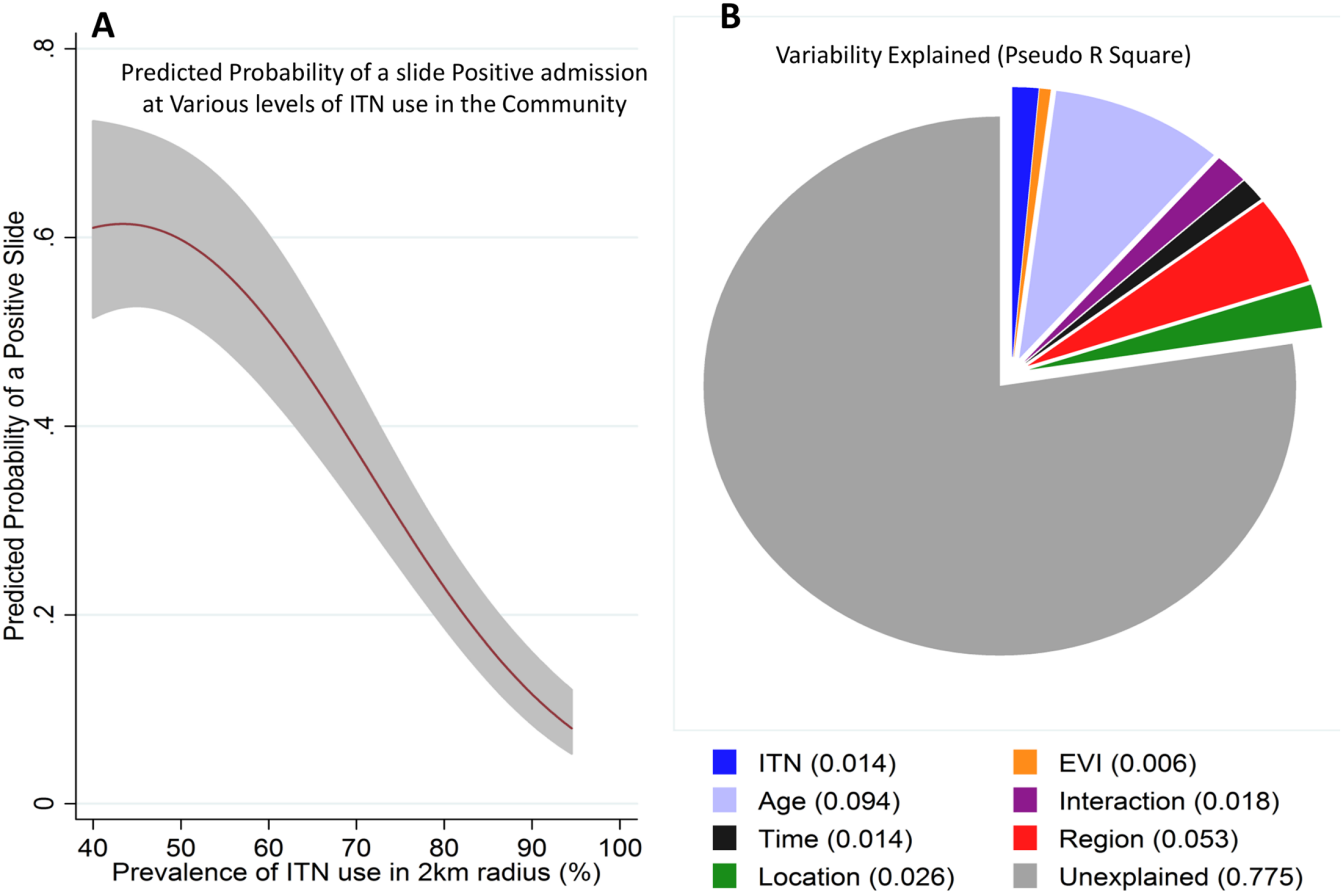
Regression model prediction and variability explained by the predictors of the model. Panel A shows the predicted probability of a positive slide result (*y*-axis) against the prevalence of ITN use in a 2 km radius around each admitted child’s residence (shaded area represents 95% CI). Panel B shows the pseudo R^2^ of the various variables assessed in the extended model (ITN use around a child’s residence, EVI, age, age–time interaction, time, region, and location). We repeated our analysis using a cut-off of >2,500 parasites per μl. The same patterns were seen—i.e., a pattern of a postdecline increase in MPF among older children (S6 Fig), and an inverse relationship between MPF and age of malaria (r = −0.63, *p* < 0.001) and a pattern of protection by community-level ITN use (S7 Fig). These patterns remained statistically significant.

The prevalence of ITN use among residents in the northern region was estimated at 32%, 16%, and 26% in the years 2000, 2003, and 2005, respectively (malaria indicator surveys, S1 Table), and then at 55.9% (95% CI 55.7–56.1) in 2009, rising to a high of 82.6% (95% CI 82.5–82.8) in 2013 in the surveys we conducted. Detailed spatial data were available between 2009 and 2014, including EVI and ITN use for each of the 250,000 residents in the study area (S4 and S5 Figs). We identified four independent predictors of malaria slide positivity: (a) ITN coverage in the 2 km radius around the child’s residence, (b) age of the child, (c) EVI in the 0.25 x 0.25 km square around the child (i.e., the finest resolution at which EVI was available), and (d) time (Fig 5B, S2 Table). Personal ITN use was a predictor in univariable analysis (odds ratio [OR] = 0.73, 95% CI 0.65–0.83, *p* < 0.001), but not after adjusting for ITN use in the 2 km radius around the admitted children’s residences (S2 Table). There were no marked differences in characteristics of missing ITN data compared to nonmissing data by age, EVI, gender, and location of residence (S3 Table). Results from multiple imputation were not significantly different from complete case analysis (S2 Table).

There was a strongly significant (*p* < 0.001) nonlinear relationship between community ITN use in the 2 km radius area around a child’s residence and a slide-positive result, such that MPF reduced substantially with increasing ITN use (Fig 5A). Similar results were obtained using a model that included clustering at 250 m^2^ (S4 Table).

## Discussion

We analyzed 69,104 hospital admissions at Kilifi County Hospital over 25 y to describe trends of malaria admissions by age group and examined the effectiveness of community ITN use. The proportion of acutely unwell children admitted to hospital with malaria-positive slides compared to all acute admissions (i.e., the MPF, also termed the “slide-positive rate”) declined from 2002 until 2009 as reported previously [19]. However, after 2009 there was an increase in MPF that continued through to 2014. This increase was mostly seen in older children: there were relatively modest increases among children less than 1 y of age (MPF_<1yr_) but much greater increases among children above 1 y of age (MPF_>1yr_). This combination of findings suggests that the increase in malaria is due to increasing susceptibility to disease among older children, exacerbated by a slight increase in underlying transmission intensity since 2009. Additional evidence for increasing transmission after 2009 can be seen in the increasing prevalence of asymptomatic parasitemia (PfPR) among children admitted for trauma and work showing increasing parasite prevalence throughout the Kenyan Coast [20].

Not all children admitted to hospital meet the criteria for severe malaria [21]. However, children admitted to hospital have higher parasite counts than children treated in the community by primary health care facilities, which likely reflects increased illness severity compared with cases treated in the community [12,22]. Previous analyses suggest that the presence of comorbidity does not necessarily indicate that the parasitemia is co-incident and not the proximate cause of admission [12,22], and we have therefore included all slide-positive admissions apart from those for trauma and elective surgery in our analysis. This analysis inevitably includes some cases of co-incidental asymptomatic parasitemia. Since asymptomatic parasitemia tends to be lower in density than the parasitemia associated with acute illness, we included a sensitivity analysis with a threshold parasite density of >2,500 parasites per μl to examine the impact of excluding asymptomatic parasitemia on our results.

It has been shown previously that at high transmission intensities, children acquire immunity rapidly and so are not susceptible to disease when they are older [6]. On the other hand, at low transmission there is less disease among younger children, and consequently, older children acquire less immunity and remain susceptible [6]. Therefore, we predict that the lowest malaria rates will occur just after a recent reduction in transmission: at this point, current exposure is low, but older children are immune because of their previous exposure when transmission was high. As time goes by after a reduction in transmission, a cohort of older children will emerge who are susceptible to the disease, and the rates of malaria will increase again.

Taking together our finding of (a) trends of increasing age of children admitted with positive malaria slides in parallel with falling PfPR_2-10_ in the community and falling MPF_<1_ among hospital admissions, (b) a geographical association in which areas with high MPF have a low average age of children admitted with malaria, and (c) previous analyses [23] and geographical analyses [4] showing the importance of the relationship between prior exposure and immunity to malaria, the most likely conclusion is that reduced exposure led to increasing susceptibility among older children and hence the subsequent increase in MPF.

Other potential explanations for increasing malaria admissions among older children could include increased prevalence of preferential ITN use among younger children rather than loss of immunity. However, multivariable models (complete case and imputed data analysis) that accounted for personal ITN use showed that age, and the interaction between age and year, were independent predictors of malaria, indicating that the increasing MPF among older children was independent of variation in ITN use by age (S2 Table). Furthermore, the difference in ITN use by age was modest (87% of children less than 1 y of age used ITNs compared with 78% of children between 1 and 5 y of age). Other interventions to prevent malaria targeted at younger children that increased over time could produce a similar pattern, but we are unaware of any interventions meeting these criteria. An increasing prevalence of outdoor biting might also preferentially affect older children. However, this would not explain the clear inverse correlations seen between MPF and average age over time and by geographical region, which suggests that immunity is an important component of the variation in MPF. In the absence of host immunity, older children may be at higher risk than younger children since mosquito biting may be proportional to surface area [24]. However, MPFs do not necessarily reflect incidence. For instance, the risk of nonmalarial fever falls as children get older, which would lead to an apparent increase in MPF.

The scale-up in ITN use occurred in 2006 and therefore is not related to the reductions in transmission seen from 2000 onwards: changing antimalarial drug use and rainfall may be relevant [20]. Regardless of the cause of the prior decline, we found clear evidence of ITN efficacy during the period in which they were used. Personal ITN use was associated with reduced MPF among children admitted to hospital. However, when multivariable models were examined, it was the prevalence of ITN use in the 2 km radius area around a child that independently reduced the probability of a malaria-positive slide, regardless of personal use. Hence, it is likely that the resurgence in malaria seen since 2009 would have been even more marked in the absence of ITNs.

The WHO and Kenyan national policy recommend universal ITN coverage to control malaria. However, operational constraints often do not allow this, and thus, many distribution strategies instead target younger children and pregnant women as high-risk groups. Our data suggest that universal coverage should be a high priority since (a) older children are increasingly in need of protection as transmission falls and (b) the mass-protective effect would achieve substantial gains in malaria control above those achieved by personal ITN use.

Fortunately, we did not identify strong evidence of an increasing case fatality rate as transmission fell (Fig 2D). The risk of mortality from severe malaria increases in older age groups across the range from children to adults [25], but this increase may be less evident within the age range of children below 10 y of age [21,26]. Furthermore, many other factors influence mortality over time, including variation in hospital use over time, access to care (and therefore earlier presentations), better care [27,28], and socioeconomic conditions. Our data do not suggest that increasing mortality is an immediate concern of the postdecline period despite the increasing rates of malaria in older children.

Study limitations include the use of MPF among acute admissions, which may be biased by varying access to care. In support of the use of MPF, we found a close correlation between location-matched MPFs with the incidence rates from active case detection data from two cohort studies conducted within the same study area. Furthermore, we showed that geographic heterogeneity was larger than any consistent bias in MPF with regards to distance to the hospital (S2 Fig), agreeing with previous work [17].

We based our analysis on the proportion of children with positive malaria slides. This case definition includes children with chronic asymptomatic parasitemia and coincident fever. To exclude a bias resulting from including these children, we repeated the analysis after applying a threshold of >2,500 parasites per μl, which excludes the majority of children with asymptomatic parasitemia (S6 Fig) [12].

Our ITN surveys were conducted annually and based on reported rather than observed use. The potential misclassification could lead to an underestimate of the protective effect of ITNs. During the study period, there were no other widespread interventions such as antimalarial prophylaxis or presumptive treatment that were targeted on younger children. ITN use has not been randomly assigned, and therefore, confounding could be present. If present, this would most likely dilute the effect size (i.e., ITN use would be better taken up in areas of higher malaria risk). Furthermore, observational studies of ITN use have consistently shown findings similar to those of randomized controlled trials (RCTs), suggesting that within specific communities confounding is not pronounced [29].

Our data were taken from a single geographical setting. However, there is evidence that immunity to malaria is dependent on exposure in a wide range of geographical settings [6], and therefore, our findings may be relevant more widely across Africa in settings where transmission is falling [2].

In conclusion, we show that despite substantial reductions in malaria transmission in Kilifi, residual malaria transmission has continued and older children are increasingly vulnerable to disease. As countries and regions make progress in malaria control [3], maintaining control measures will be essential: in fact, further progress will be required to offset the increasing rates of malaria in older children. Achieving ITN coverage close to 100% shows promise as a strategy but will require novel strategies, particularly among groups that seem less keen to use ITNs, such as adolescent males and young adults [30].

## Supporting Information

S1 Fig Trends of malaria positivity by age group (years) and transmission period. The graph shows the trend of MPF in each age category <1 (less than 1 y old), 1–3 (1 to 3 y old), 3–5 (3 to 4 y old), >5 (children over 5 y old).
(TIFF)

S2 Fig Association and trends of MPF with distance from hospital. The figure shows the association of MPF with distance from hospital, the green line presents the predicted linear regression line, the red line presents the predicted line from the multiple fractional polynomial model, and the blue line presents the fitted lowess function.
(TIFF)

S3 Fig Malaria incidence in active case detection compared with MPF. The figure shows the association between malaria incidence from active case detection in the Junju and Ngerenya areas and that of MPF among patients admitted to the hospital from these sites (Rho = 0.84, *p* < 0.001).
(TIFF)

S4 Fig Finer-scale geographical distribution of MPF (Row I), ITN use (Row II), and EVI (Row III) over time and the average admissions per year (Row IVa) and population density (Row IVb). Panels IVc, IVd, and IVe are the legends for Row I, Row II, and Row III, respectively.
(TIFF)

S5 Fig Trends in ITN use and EVI. Panel A shows the trends of EVI from year 2000 through to 2014. Panel B shows the trend of ITN use for a 6-y period (2009–2014).
(TIFF)

S6 Fig Trends of MPF, over time and region using a case definition for malaria of >2,500 parasites/μl. Panels A and B show the temporal trends of MPF in admitted children aged ≤ 1 y old and children aged > 1 y old, respectively; the red line represents the southern region, while the blue line represents the northern region of the hospital.
(TIFF)

S7 Fig Regression model prediction using a case definition for malaria of >2,500 parasites/μl. The figure shows the predicted probability of a positive slide result (*y*-axis) against the prevalence of ITN use in a 2 km radius around each admitted child’s residence.
(TIFF)

S1 Table Data from malaria indicator surveys conducted in the northern region of the Kilifi demographic surveillance area. (DOCX)

S2 Table Multiple logistic regression model of malaria on personal ITN use, ITN use prevalence around the child’s residence, and EVI (complete case analysis compared to multiple imputation analysis). (DOCX)

S3 Table Characteristic of individuals with missing ITN data during community ITN use surveys. (DOCX)

S4 Table Multiple logistic regression model of malaria on personal ITN use, ITN use prevalence around the child’s residence, and EVI (allowing clustering at 250 m^2^). (DOCX)

S1 STROBE Checklist STROBE checklist. (DOC)

S1 Video Shows the spatiotemporal variations of MPF in Kilifi County. (MP4)

## Abbreviations

CI: confidence interval
EVI: enhanced vegetation index
IQR: interquartile range
ITN: insecticide-treated net
KEMRI: Kenya Medical Research Institute
KHDSS: Kilifi Health and Demographic Surveillance System
MODIS: Moderate-Resolution Imaging Spectroradiometer
MPF: malaria positive fraction
NASA: National Aeronautics and Space Administration
OR: odds ratio
PfPR: *Plasmodium falciparum* parasite rate
RCT: randomized controlled trial

## References

[1] Ceesay SJ, Casals-Pascual C, Nwakanma DC, Walther M, Gomez-Escobar N, Fulford AJ, et al Continued decline of malaria in The Gambia with implications for elimination. PLoS ONE. 2010;5(8):e12242 Epub 2010/09/02. 10.1371/journal.pone.0012242 ; PubMed Central PMCID: PMCPmc2923605. 20805878 PMC2923605

[2] O'Meara WP, Mangeni JN, Steketee R, Greenwood B. Changes in the burden of malaria in sub-Saharan Africa. The Lancet Infectious Diseases. 2010;10(8):545–55. 10.1016/S1473-3099(10)70096-7 20637696

[3] Noor AM, Kinyoki DK, Mundia CW, Kabaria CW, Mutua JW, Alegana VA, et al The changing risk of Plasmodium falciparum malaria infection in Africa: 2000–10: a spatial and temporal analysis of transmission intensity. Lancet. 2014;383(9930):1739–47. Epub 2014/02/25. 10.1016/s0140-6736(13)62566-0 ; PubMed Central PMCID: PMCPmc4030588. 24559537 PMC4030588

[4] Snow RW, Omumbo JA, Lowe B, Molyneux CS, Obiero JO, Palmer A, et al Relation between severe malaria morbidity in children and level of Plasmodium falciparum transmission in Africa. Lancet. 1997;349(9066):1650–4. Epub 1997/06/07. 10.1016/s0140-6736(97)02038-2 . 9186382

[5] Woolhouse ME. Patterns in parasite epidemiology: the peak shift. Parasitol Today. 1998;14(10):428–34. Epub 2006/10/17. . 17040835 10.1016/s0169-4758(98)01318-0

[6] Okiro EA, Al-Taiar A, Reyburn H, Idro R, Berkley JA, Snow RW. Age patterns of severe paediatric malaria and their relationship to Plasmodium falciparum transmission intensity. Malaria journal. 2009;8:4 Epub 2009/01/09. 10.1186/1475-2875-8-4 ; PubMed Central PMCID: PMCPmc2630996. 19128453 PMC2630996

[7] Karema C, Aregawi MW, Rukundo A, Kabayiza A, Mulindahabi M, Fall IS, et al Trends in malaria cases, hospital admissions and deaths following scale-up of anti-malarial interventions, 2000–2010, Rwanda. Malaria journal. 2012;11:236 Epub 2012/07/25. 10.1186/1475-2875-11-236 ; PubMed Central PMCID: PMCPmc3502144. 22823945 PMC3502144

[8] Trape JF, Tall A, Diagne N, Ndiath O, Ly AB, Faye J, et al Malaria morbidity and pyrethroid resistance after the introduction of insecticide-treated bednets and artemisinin-based combination therapies: a longitudinal study. Lancet Infect Dis. 2011;11(12):925–32. Epub 2011/08/23. 10.1016/s1473-3099(11)70194-3 . 21856232

[9] Pemberton-Ross P, Smith TA, Hodel EM, Kay K, Penny MA. Age-shifting in malaria incidence as a result of induced immunological deficit: a simulation study. Malaria journal. 2015;14:287 Epub 2015/07/25. 10.1186/s12936-015-0805-1 ; PubMed Central PMCID: PMCPmc4513612. 26206255 PMC4513612

[10] Midekisa A, Senay G, Henebry GM, Semuniguse P, Wimberly MC. Remote sensing-based time series models for malaria early warning in the highlands of Ethiopia. Malaria journal. 2012;11:165 Epub 2012/05/16. 10.1186/1475-2875-11-165 ; PubMed Central PMCID: PMCPmc3493314. 22583705 PMC3493314

[11] Scott JA, Bauni E, Moisi JC, Ojal J, Gatakaa H, Nyundo C, et al Profile: The Kilifi Health and Demographic Surveillance System (KHDSS). Int J Epidemiol. 2012;41(3):650–7. Epub 2012/05/01. 10.1093/ije/dys062 ; PubMed Central PMCID: PMCPmc3396317. 22544844 PMC3396317

[12] Bejon P, Berkley JA, Mwangi T, Ogada E, Mwangi I, Maitland K, et al Defining childhood severe falciparum malaria for intervention studies. PLoS Med. 2007;4(8):e251 Epub 2007/08/24. 10.1371/journal.pmed.0040251 ; PubMed Central PMCID: PMCPmc1949845. 17713980 PMC1949845

[13] Mwangi TW, Ross A, Snow RW, Marsh K. Case definitions of clinical malaria under different transmission conditions in Kilifi District, Kenya. J Infect Dis. 2005;191(11):1932–9. . 15871128 10.1086/430006 PMC3545188

[14] Smith DL, Guerra CA, Snow RW, Hay SI. Standardizing estimates of the Plasmodium falciparum parasite rate. Malaria journal. 2007;6:131 Epub 2007/09/27. 10.1186/1475-2875-6-131 ; PubMed Central PMCID: PMCPmc2072953. 17894879 PMC2072953

[15] Tatem AJ, Goetz SJ, Hay SI. Terra and Aqua: new data for epidemiology and public health. Int J Appl Earth Obs Geoinf. 2004;6(1):33–46. Epub 2004/11/01. ; PubMed Central PMCID: PMCPmc3337546. 22545030 10.1016/j.jag.2004.07.001 PMC3337546

[16] Team RC. R: A language and environment for statistical computing. R Foundation for Statistical Computing, Vienna, Austria 2013.

[17] Bejon P, Williams TN, Nyundo C, Hay SI, Benz D, Gething PW, et al A micro-epidemiological analysis of febrile malaria in Coastal Kenya showing hotspots within hotspots. Elife. 2014;3:e02130 Epub 2014/05/21. 10.7554/eLife.02130 ; PubMed Central PMCID: PMCPmc3999589. 24843017 PMC3999589

[18] Snow RW, Molyneux C, Warn P, Omumbo J, Nevill C, Gupta S, et al Infant parasite rates and immunoglobulin M seroprevalence as a measure of exposure to Plasmodium falciparum during a randomized controlled trial of insecticide-treated bed nets on the Kenyan coast. Am J Trop Med Hyg. 1996;55(2):144–9. 8780451

[19] O'Meara WP, Bejon P, Mwangi TW, Okiro EA, Peshu N, Snow RW, et al Effect of a fall in malaria transmission on morbidity and mortality in Kilifi, Kenya. Lancet. 2008;372(9649):1555–62. Epub 2008/11/06. 10.1016/s0140-6736(08)61655-4 ; PubMed Central PMCID: PMCPmc2607008. 18984188 PMC2607008

[20] Snow RW, Kibuchi E, Karuri SW, Sang G, Gitonga CW, Mwandawiro C, et al Changing Malaria Prevalence on the Kenyan Coast since 1974: Climate, Drugs and Vector Control. PLoS ONE. 2015;10(6):e0128792 Epub 2015/06/25. 10.1371/journal.pone.0128792 . 26107772 PMC4479373

[21] Marsh K, Forster D, Waruiru C, Mwangi I, Winstanley M, Marsh V, et al Indicators of life-threatening malaria in African children. The New England journal of medicine. 1995;332(21):1399–404. Epub 1995/05/25. 10.1056/nejm199505253322102 . 7723795

[22] Goncalves BP, Huang CY, Morrison R, Holte S, Kabyemela E, Prevots DR, et al Parasite burden and severity of malaria in Tanzanian children. The New England journal of medicine. 2014;370(19):1799–808. Epub 2014/05/09. 10.1056/NEJMoa1303944 ; PubMed Central PMCID: PMCPmc4091983. 24806160 PMC4091983

[23] Reyburn H, Mbatia R, Drakeley C, Bruce J, Carneiro I, Olomi R, et al Association of transmission intensity and age with clinical manifestations and case fatality of severe Plasmodium falciparum malaria. Jama. 2005;293(12):1461–70. . 15784869 10.1001/jama.293.12.1461

[24] Smith T, Killeen G, Lengeler C, Tanner M. Relationships between the outcome of Plasmodium falciparum infection and the intensity of transmission in Africa. The American journal of tropical medicine and hygiene. 2004;71(2 Suppl):80–6. Epub 2004/08/28. . 15331822

[25] Dondorp AM, Lee SJ, Faiz MA, Mishra S, Price R, Tjitra E, et al The relationship between age and the manifestations of and mortality associated with severe malaria. Clin Infect Dis. 2008;47(2):151–7. 10.1086/589287 . 18533842

[26] von Seidlein L, Olaosebikan R, Hendriksen IC, Lee SJ, Adedoyin OT, Agbenyega T, et al Predicting the clinical outcome of severe falciparum malaria in african children: findings from a large randomized trial. Clin Infect Dis. 2012;54(8):1080–90. 10.1093/cid/cis034 22412067 PMC3309889

[27] Maitland K, Kiguli S, Opoka RO, Engoru C, Olupot-Olupot P, Akech SO, et al Mortality after fluid bolus in African children with severe infection. The New England journal of medicine. 2011;364(26):2483–95. Epub 2011/05/28. 10.1056/NEJMoa1101549 . 21615299

[28] Dondorp AM, Fanello CI, Hendriksen IC, Gomes E, Seni A, Chhaganlal KD, et al Artesunate versus quinine in the treatment of severe falciparum malaria in African children (AQUAMAT): an open-label, randomised trial. Lancet. 2010;376(9753):1647–57. Epub 2010/11/11. 10.1016/s0140-6736(10)61924-1 ; PubMed Central PMCID: PMCPmc3033534. 21062666 PMC3033534

[29] Lim SS, Fullman N, Stokes A, Ravishankar N, Masiye F, Murray CJ, et al Net benefits: a multicountry analysis of observational data examining associations between insecticide-treated mosquito nets and health outcomes. PLoS Med. 2011;8(9):e1001091 Epub 2011/09/13. 10.1371/journal.pmed.1001091 ; PubMed Central PMCID: PMCPmc3167799. 21909249 PMC3167799

[30] Noor AM, Kirui VC, Brooker SJ, Snow RW. The use of insecticide treated nets by age: implications for universal coverage in Africa. BMC Public Health. 2009;9:369 Epub 2009/10/03. 1471-2458-9-369 [pii] 10.1186/1471-2458-9-369 19796380 PMC2761895

