# Supplementary figures and images for: Age, Spatial, and Temporal Variations in Hospital Admissions with Malaria in Kilifi County, Kenya: A 25-Year Longitudinal Observational Study
Source: PLoS Med. 2016 Jun 28;13(6):e1002047. doi: 10.1371/journal.pmed.1002047 (PMC4924798; doi:10.1371/journal.pmed.1002047)

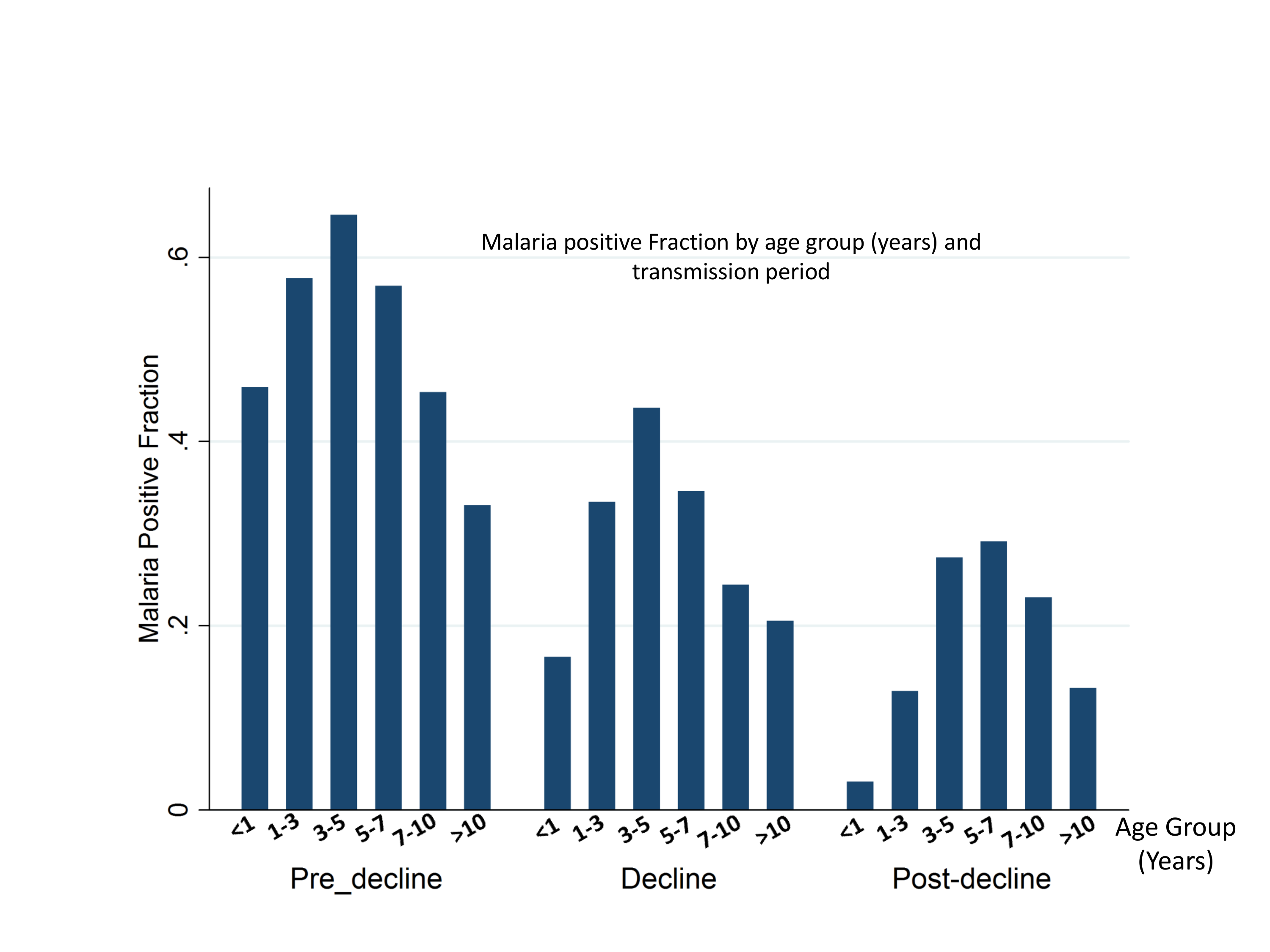

Supplement: S1 Fig — The graph shows the trend of MPF in each age category <1 (less than 1 y old), 1–3 (1 to 3 y old), 3–5 (3 to 4 y old), >5 (children over 5 y old). (TIFF) [file pmed.1002047.s001.tiff]

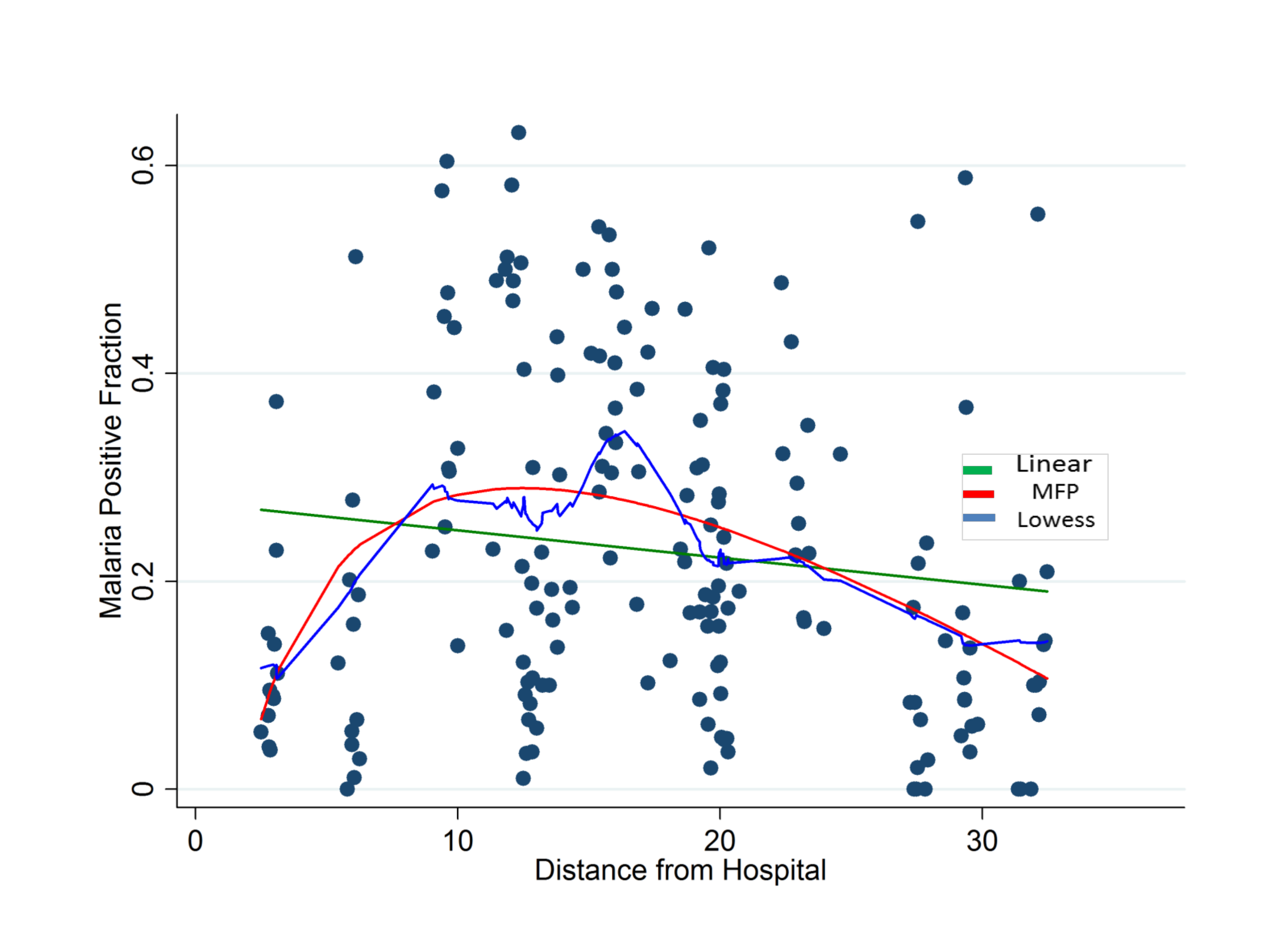

Supplement: S2 Fig — The figure shows the association of MPF with distance from hospital, the green line presents the predicted linear regression line, the red line presents the predicted line from the multiple fractional polynomial model, and the blue line presents the fitted lowess function. (TIFF) [file pmed.1002047.s002.tiff]

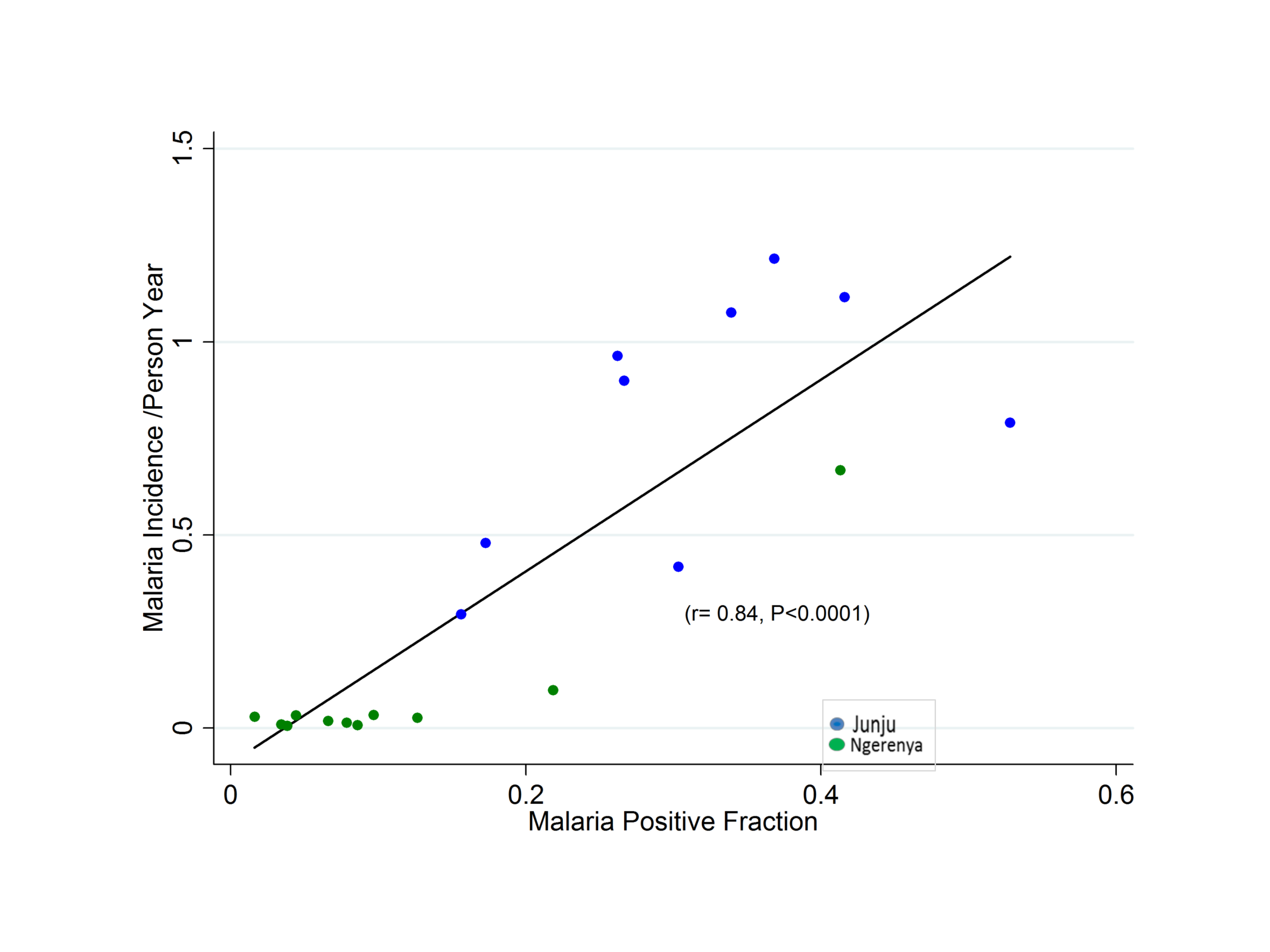

Supplement: S3 Fig — The figure shows the association between malaria incidence from active case detection in the Junju and Ngerenya areas and that of MPF among patients admitted to the hospital from these sites (Rho = 0.84, p < 0.001). (TIFF) [file pmed.1002047.s003.tiff]

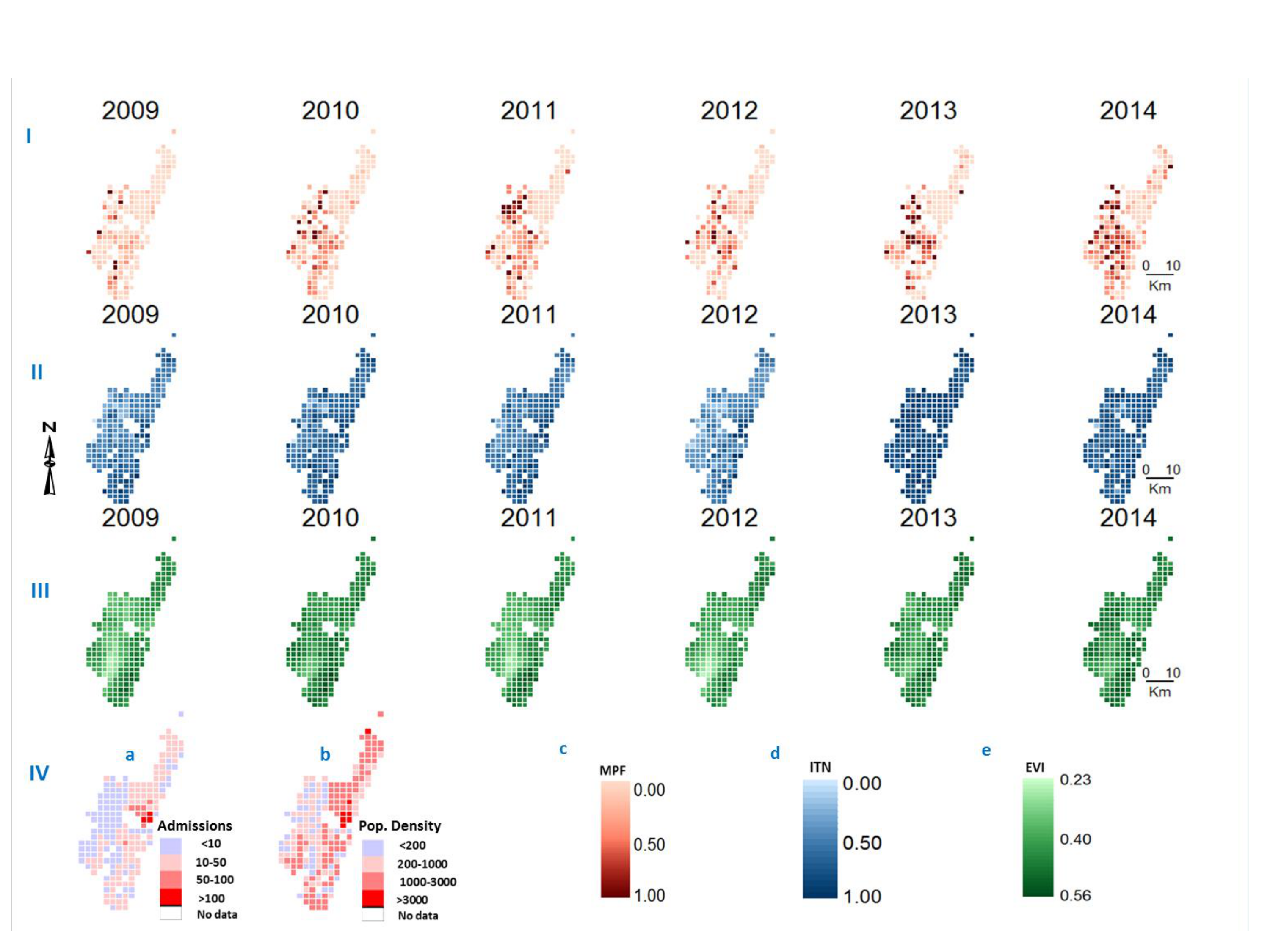

Supplement: S4 Fig — Panels IVc, IVd, and IVe are the legends for Row I, Row II, and Row III, respectively. (TIFF) [file pmed.1002047.s004.tiff]

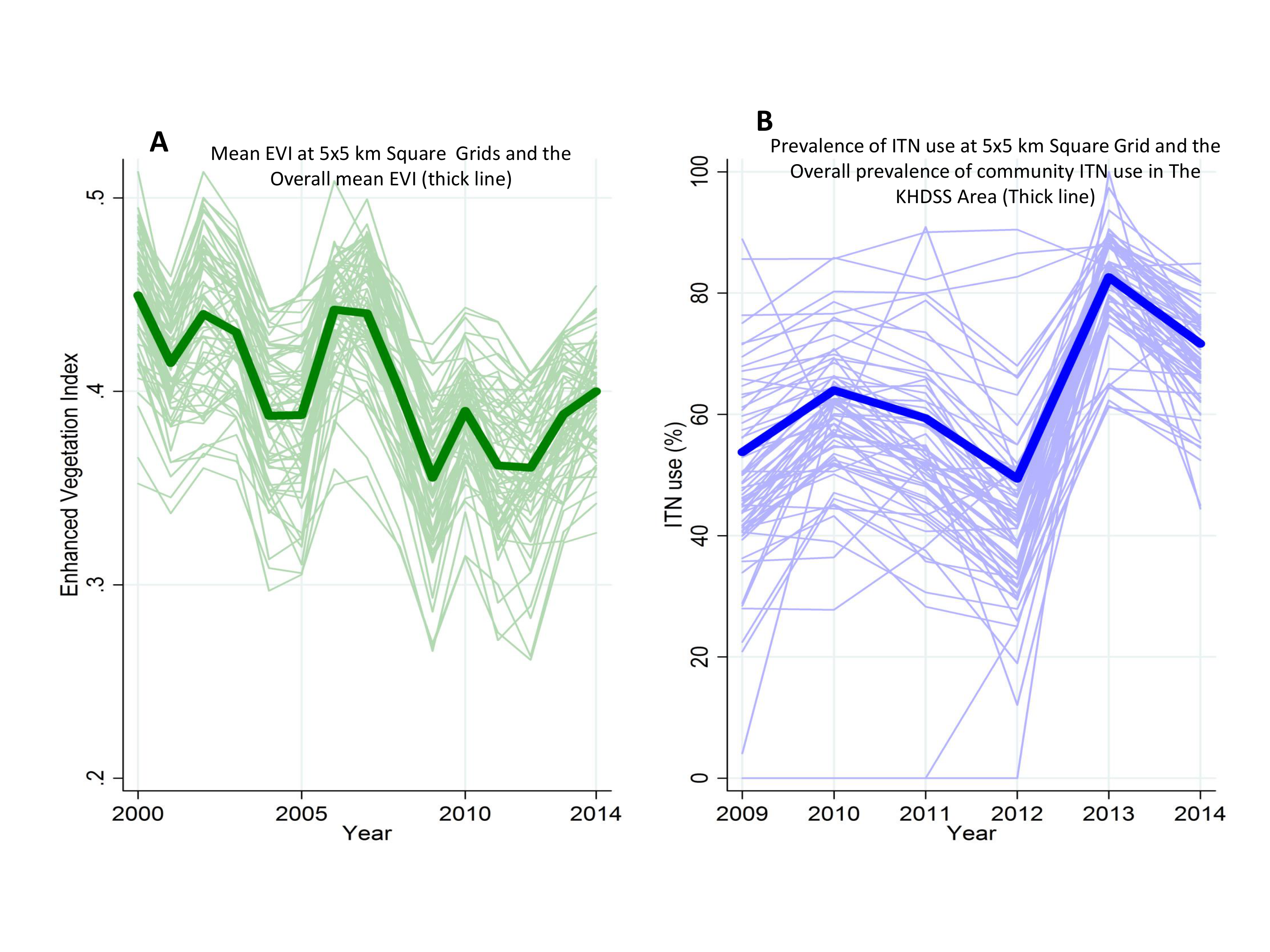

Supplement: S5 Fig — Panel A shows the trends of EVI from year 2000 through to 2014. Panel B shows the trend of ITN use for a 6-y period (2009–2014). (TIFF) [file pmed.1002047.s005.tiff]

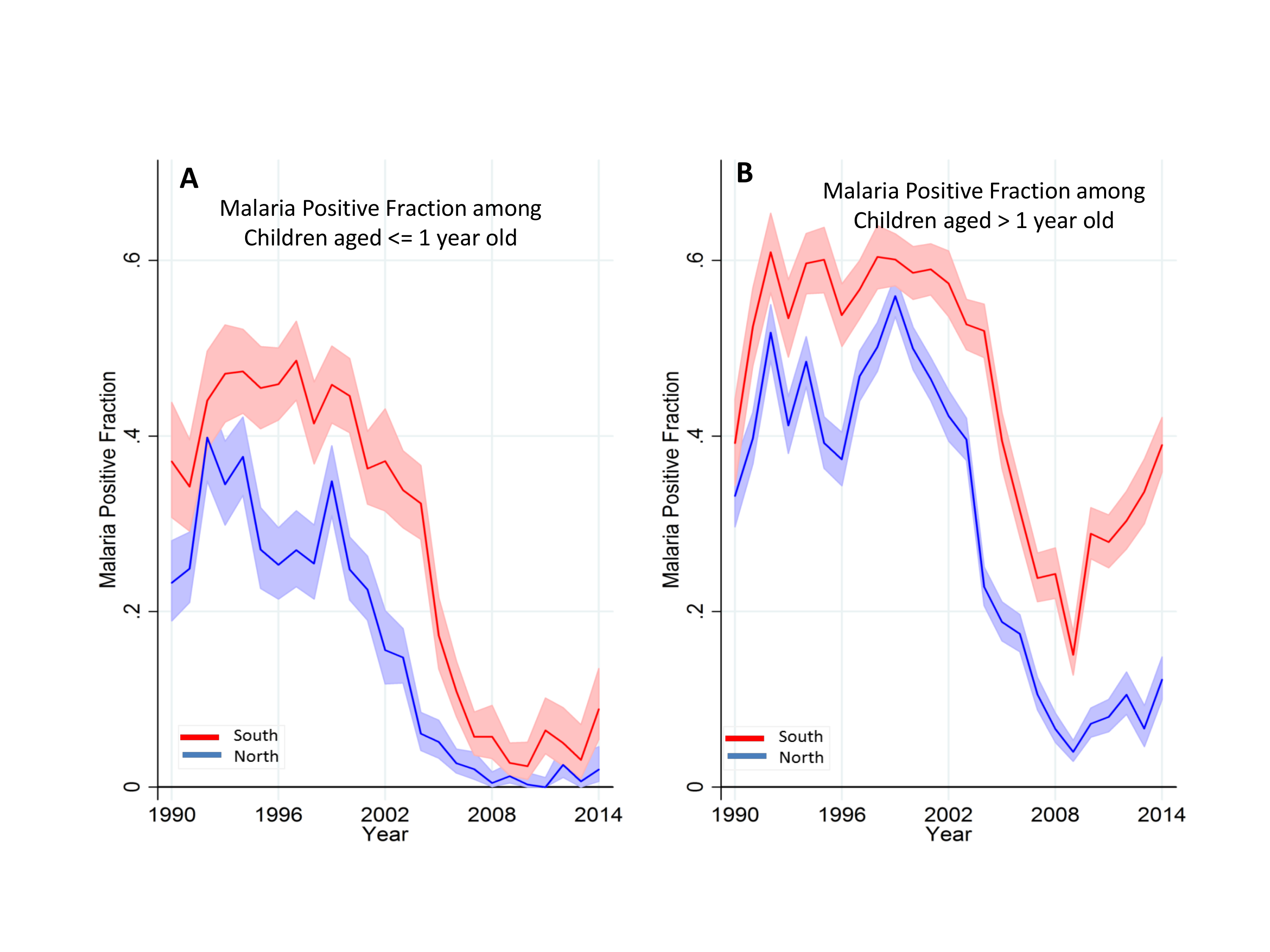

Supplement: S6 Fig — Panels A and B show the temporal trends of MPF in admitted children aged ≤ 1 y old and children aged > 1 y old, respectively; the red line represents the southern region, while the blue line represents the northern region of the hospital. (TIFF) [file pmed.1002047.s006.tiff]

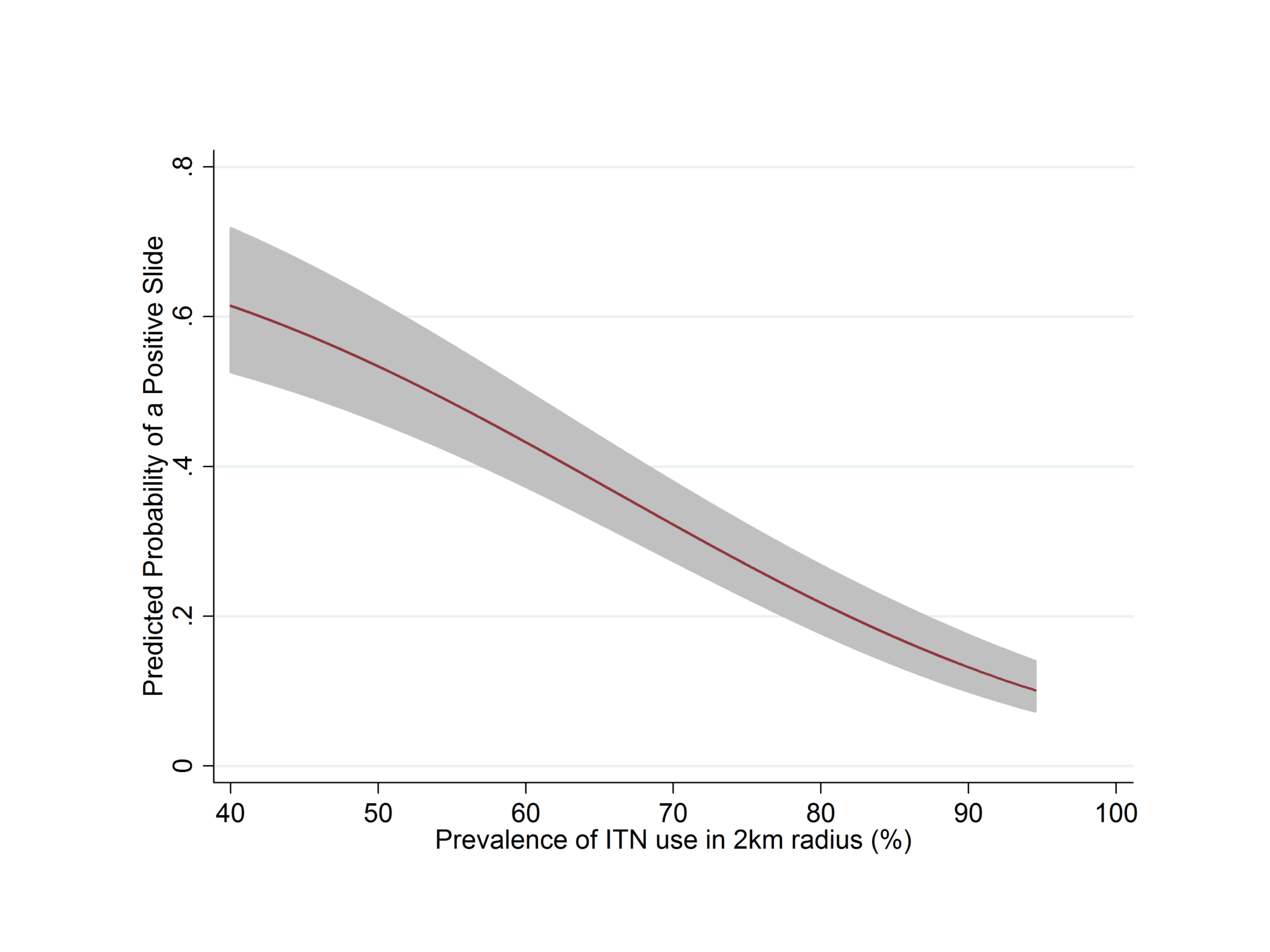

Supplement: S7 Fig — The figure shows the predicted probability of a positive slide result (y-axis) against the prevalence of ITN use in a 2 km radius around each admitted child’s residence. (TIFF) [file pmed.1002047.s007.tiff]
