# Supplementary material for: Age, Spatial, and Temporal Variations in Hospital Admissions with Malaria in Kilifi County, Kenya: A 25-Year Longitudinal Observational Study
Source: PLoS Med. 2016 Jun 28;13(6):e1002047. doi: 10.1371/journal.pmed.1002047 (PMC4924798; doi:10.1371/journal.pmed.1002047)
Supplement: S1 Table — (DOCX) [file pmed.1002047.s008.docx]

| Clusterid | Year | population size | ITN use (N) |
| --- | --- | --- | --- |
| 157 | 2000 | 19 | 10 |
| 159 | 2000 | 11 | 11 |
| 160 | 2000 | 11 | 2 |
| 161 | 2000 | 11 | 0 |
| 162 | 2000 | 16 | 5 |
| 163 | 2000 | 16 | 4 |
| 164 | 2000 | 10 | 0 |
| 165 | 2000 | 13 | 1 |
| 166 | 2000 | 7 | 2 |
| 167 | 2000 | 13 | 2 |
| 168 | 2000 | 7 | 4 |
| 170 | 2000 | 8 | 0 |
| 172 | 2000 | 14 | 4 |
| 173 | 2000 | 11 | 9 |
| 174 | 2000 | 16 | 1 |
| 177 | 2000 | 17 | 3 |
| 179 | 2000 | 10 | 5 |
| 180 | 2000 | 12 | 7 |
| 1230 | 2000 | 12 | 4 |
| 2 | 2003 | 87 | 39 |
| 48 | 2003 | 118 | 0 |
| 86 | 2003 | 113 | 3 |
| 99 | 2003 | 54 | 27 |
| 279 | 2003 | 99 | 5 |
| 280 | 2003 | 129 | 15 |
| 286 | 2003 | 66 | 22 |
| 291 | 2003 | 43 | 7 |
| 317 | 2003 | 114 | 21 |
| 336 | 2003 | 112 | 22 |
| 349 | 2003 | 73 | 31 |
| 359 | 2003 | 99 | 0 |
| 362 | 2003 | 156 | 11 |
| 163 | 2005 | 33 | 22 |
| 167 | 2005 | 28 | 7 |
| 171 | 2005 | 53 | 7 |
| 176 | 2005 | 51 | 14 |
| 180 | 2005 | 68 | 4 |
| 259 | 2005 | 95 | 16 |
| 265 | 2005 | 81 | 6 |
| 271 | 2005 | 59 | 29 |
| 1418 | 2005 | 37 | 17 |
| 1478 | 2005 | 29 | 11 |
| 1482 | 2005 | 21 | 12 |
