# Supplementary material for: Age, Spatial, and Temporal Variations in Hospital Admissions with Malaria in Kilifi County, Kenya: A 25-Year Longitudinal Observational Study
Source: PLoS Med. 2016 Jun 28;13(6):e1002047. doi: 10.1371/journal.pmed.1002047 (PMC4924798; doi:10.1371/journal.pmed.1002047)
Supplement: S2 Table — (DOCX) [file pmed.1002047.s009.docx]

|  | | **Complete Case Analysis** | | | **Imputed data Analysis** | | |
| --- | --- | --- | --- | --- | --- | --- | --- |
| **Covariates** | **Covariates From Multiple Fractional Polynomial model** | **Odds Ratio** | **P-value** | **(95% CI)** | **Odds Ratio** | **P-value** | **(95% CI)** |
| Personal ITN use | Personal ITN use | 0.925 | 0.307 | (0.797 1.075) | 0.937 | 0.37 | (0.814 1.087) |
| Community level ITN use (2km radius) | ITN1=ITN_2km^2-0.3908547648 | 0.159 | <0.001 | (0.104 0.242) | 0.154 | <0.001 | (0.102 0.233) |
|  | ITN2=ITN_2km^2*ln(ITN_2km) +0.1835882418 | 2.3E-4 | <0.001 | (1.1E-5 5.0E-3) | 4.9E-4 | <0.001 | (3.0E-5 8.4E-3) |
| Age in Years | Age1=(Age/10)^0.5-0.5888197049 | 3.0E+07 | <0.001 | (4.6E+6 1.9E+8) | 1.3E+07 | <0.001 | (2.6E+6 6.6E+7) |
|  | Age2=(Age/10)-0.3467086449 | 6.1E-06 | <0.001 | (1.5E-6 2.5E-5) | 2.8E-05 | <0.001 | (8.9E-6 8.6E-5) |
| EVI (0.25x0.25 km) | EVI | 23.43 | <0.001 | (4.40 124.17) | 12.26 | 0.001 | (2.73 55.00) |
| Time=(year-2008) | T1=ln(time)-1.142973262 | 2.11 | <0.001 | (1.77 2.43) | 1.30 | <0.001 | (1.24 1.36) |
| Age-Time interaction | Age1*T1 | 4.2E-3 | 0.002 | (1.3E-4 0.14) | 0.32 | 0.01 | (0.14 0.76) |
|  | Age2*T1 | 120.56 | <0.001 | (8.3 1.8E+3) | 2.44 | 0.003 | (1.35 4.40) |
