# Supplementary material for: Age, Spatial, and Temporal Variations in Hospital Admissions with Malaria in Kilifi County, Kenya: A 25-Year Longitudinal Observational Study
Source: PLoS Med. 2016 Jun 28;13(6):e1002047. doi: 10.1371/journal.pmed.1002047 (PMC4924798; doi:10.1371/journal.pmed.1002047)
Supplement: S3 Table — (DOCX) [file pmed.1002047.s010.docx]

| Characteristics | **Missing data** | **Complete data** |
| --- | --- | --- |
| Overall No. (%) | 190,411 (12.81) | 1,296,346 (87.19) |
| Age (years) median (iqr) | 19 (8 - 31) | 15 (7 - 32) |
| EVI median (iqr) | 0.366 (0.326-0.393) | 0.370(0.336-0.397) |
| Male No. (%) | 75,823 (48.16) | 501,641 (46.70) |
| **Missing by locations** |  |  |
| Chonyi No. (%) | 28,178 (14.80) | 215,829 (16.65) |
| Gede No. (%) | 14,649 (7.69) | 108,598 (8.38) |
| Jaribuni No. (%) | 7,904 (4.15) | 70,616 (5.45) |
| Junju No. (%) | 23,470 (12.33) | 150,654 (11.62) |
| Mtwapa No. (%) | 9,870 (5.18) | 63,074 (4.87) |
| Ngerenya No. (%) | 9,834 (5.16) | 79,737 (6.15) |
| Roka No. (%) | 11,714 (6.15) | 87,215 (6.73) |
| Sokoke No. (%) | 5,251 (2.76) | 44,089 (3.40) |
| Takaungu No. (%) | 16,751 (8.80) | 137,710 (10.62) |
| Tezo No. (%) | 62,790 (32.98) | 338,822 (26.14) |
