# Supplementary material for: Age, Spatial, and Temporal Variations in Hospital Admissions with Malaria in Kilifi County, Kenya: A 25-Year Longitudinal Observational Study
Source: PLoS Med. 2016 Jun 28;13(6):e1002047. doi: 10.1371/journal.pmed.1002047 (PMC4924798; doi:10.1371/journal.pmed.1002047)
Supplement: S4 Table — (DOCX) [file pmed.1002047.s011.docx]

| **Covariates** | **Covariates From Multiple Fractional Polynomial model** | **Odds Ratio** | **P-value** | **(95% CI)** |
| --- | --- | --- | --- | --- |
| Personal ITN use | Personal ITN use | 0.925 | 0.355 | (0.78 1.09) |
| Community level ITN use (2km radius) | ITN1=ITN_2km^2-0.3908547648 | 0.159 | <0.001 | (0.097 0.259) |
|  | ITN2=ITN_2km^2*ln(ITN_2km) +0.1835882418 | 2.3E-4 | <0.001 | (6.6E-6 0.008) |
| Age in Years | Age1=(Age/10)^0.5-0.5888197049 | 3.0E+07 | <0.001 | (4.0E+6 2.3E+8) |
|  | Age2=(Age/10)-0.3467086449 | 6.1E-06 | <0.001 | (1.3E-6 2.9E-5) |
| EVI (0.25x0.25 km) | EVI | 23.4 | <0.003 | (2.86 190.7) |
| Time=(year-2008) | T1=ln(time)-1.142973262 | 2.1 | <0.001 | (1.76 2.44) |
| Age-Time interaction | Age1*T1 | 4.2E-3 | 0.004 | (1.0E-4 0.17) |
|  | Age2*T1 | 120.56 | 0.001 | (7.1 2.0E+3) |
