# Supplementary material for: Age, Spatial, and Temporal Variations in Hospital Admissions with Malaria in Kilifi County, Kenya: A 25-Year Longitudinal Observational Study
Source: PLoS Med. 2016 Jun 28;13(6):e1002047. doi: 10.1371/journal.pmed.1002047 (PMC4924798; doi:10.1371/journal.pmed.1002047)
Supplement: S1 STROBE Checklist — (DOC) [file pmed.1002047.s012.doc]

STROBE Statement—checklist of items that should be included in reports of observational studies

|  | Item No | Recommendation |
| --- | --- | --- |
| **Title and abstract** | 1 | (*a*) Indicate the study’s design with a commonly used term in the title or the abstract  >“Longitudinal Observational Study” |
|  |  | (*b*) Provide in the abstract an informative and balanced summary of what was done and what was found  *>The manuscript abstract contains a summary of what was done and found as follows;*  “The variation in malaria slide positivity among admissions was examined in logistic regression models using the predictors location of residence, time, child’s age, ITN use and Enhanced Vegetation Index (a proxy for soil moisture).  The proportion of malaria slide positive admissions declined from 0.56 with 95% confidence interval (95%CI) 0.54 to 0.58 in 1998 to 0.07 95%CI 0.06 to 0.08 in 2009, but then increased again through to 0.24 95%CI 0.22 to 0.25 in 2014. Older children accounted for most of the increase after 2009 (0.035 95%CI (0.03 to 0.04) among young children compared to 0.22 95%CI 0.21 to 0.23 in older children). The predicted  malaria positive fraction varied from ~0.4 to <0.1 as the prevalence of ITN use varied from 20% to 80%.” |
| Introduction | | |
| Background/rationale | 2 | Explain the scientific background and rationale for the investigation being reported  *>The scientific background and rationale was discussed in the Introduction as follows;*  “Reduced transmission intensity of infectious diseases has been associated with increasing age of susceptibility [1]. This trend has been reported in malaria in both field conditions [2-4] and simulation studies incorporating acquired immunity [5]. For instance reductions in malaria transmission led to more marked improvements in outcomes for children under 5 years than in the older age groups in Rwanda [3]. In Senegal, a rebound in malaria attacks was observed among adults and older children following a period of declining transmission [4]. These observations may be attributed to reduced population-levels of immunity due to reduced exposure. It is therefore essential to monitor outcomes following initial reductions in malaria transmission.” |
| Objectives | 3 | State specific objectives, including any prespecified hypotheses  *>The objectives were stated in the Manuscript as follows;*  “The objective of the analysis was to describe trends of malaria admissions by age group and examine the effectiveness of community Insecticide Treated Nets (ITN) use.” |
| Methods | | |
| Study design | 4 | Present key elements of study design early in the paper  *>Key study design elements are stated in the Manuscript as follows;*  ‘Screening for malaria parasites has been continuous for 25 years since the establishment of a pediatric admission ward surveillance system in 1989.” |
| Setting | 5 | Describe the setting, locations, and relevant dates, including periods of recruitment, exposure, follow-up, and data collection  *>The Study setting, locations, and relevant dates and data collection were stated in the Manuscript as follows;*  “The study was conducted at Kilifi County Hospital which is situated at the center of the Kilifi Health and Demographic Surveillance System (KHDSS) area. The KHDSS covers a population of ~260,000 people living in an area of ~891 km^2^, as described previously [6]. Screening for malaria parasites has been continuous for 25 years since the establishment of a pediatric ward surveillance system in 1989.  Children below 13 years of age were admitted to the pediatric service, where demographic details and clinical history were recorded and blood was examined for malaria parasites by microscopy. These assessments were conducted on all emergency admissions, irrespective of presumptive diagnosis, with the exception of those admitted for elective surgery." |
| Participants | 6 | (*a*) *Cohort study*—Give the eligibility criteria, and the sources and methods of selection of participants. Describe methods of follow-up  Not Applicable  *Case-control study*—Give the eligibility criteria, and the sources and methods of case ascertainment and control selection. Give the rationale for the choice of cases and controls  *Eligibility criteria, sources and methods of case ascertainment, control selection and the rationale for the choice of cases and controls are discussed in the manuscript as follows*  “Children admitted to Kilifi County Hospital who were aged from 3 months to 13 years between 1990 and 2014 and were residents of the KHDSS area.  Children below 13 years of age were admitted to the pediatric service, where demographic details and clinical history were recorded and blood was examined for malaria parasites by microscopy. These assessments were conducted on all emergency admissions, irrespective of presumptive diagnosis, with the exception of those admitted for elective surgery. “Malaria” included all acute admissions with positive slides.”  *Cross-sectional study*—Give the eligibility criteria, and the sources and methods of selection of participants  Not Applicable |
|  |  | (*b*) *Cohort study*—For matched studies, give matching criteria and number of exposed and unexposed  Not Applicable  *Case-control study*—For matched studies, give matching criteria and the number of controls per case  Not Applicable |
| Variables | 7 | Clearly define all outcomes, exposures, predictors, potential confounders, and effect modifiers. Give diagnostic criteria, if applicable  *>Outcomes, exposures, predictors were defined as follows;*  “Presence or absence of malaria parasites by microscopy among medical admissions (excluding trauma and elective surgery) as the outcome variable, time (continuous variable to capture the secular trends), year (categorical to capture year-to-year variability), region (referring to North vs South of the creek), location (referring to administrative areas within region) and space-time interactions as the independent covariates.”  More description on ITN use and EVI is given in the Covariate data section of the manuscript |
| Data sources/ measurement | 8* | For each variable of interest, give sources of data and details of methods of assessment (measurement). Describe comparability of assessment methods if there is more than one group  *>The outcome variable* “malaria (case/control)” was derived from the Kilifi County Hospital Admission Surveillance System.  “Malaria case included all acute admissions with positive slides by microscopy.” Malaria controls included all acute admissions with negative slides by microscopy.”  *>EVI was extracted from NASA’s Archives and was described in the paper as follows;* “Enhanced Vegetation Index (EVI) data available between 2000 and 2014 were used as a proxy for soil moisture [7]. They were derived from NASA’s remote sensing Moderate Resolution Imaging Spectroradiometer (MODIS) instrument on board the Terra satellites [8]”  *>ITN use data was collected during the survey in the community and is described in the paper as follows:-*  Data on ITN use were obtained from yearly KHDSS surveys of all residents conducted from 2009 through 2014. We used data from responses to the question “did you sleep under an ITN last night?” or “did your child sleep under an ITN last night?” |
| Bias | 9 | Describe any efforts to address potential sources of bias  >*Bias due to Distance to Hospital from the residence area was thought to be a potential source of bias, this was examined and described in the paper as follows;*  “In order to assess the effect of access to care, we examined the data for trends in MPF over the euclidean distance between children’s residence and the hospital (S2 Fig). Best-fit lines showed greater heterogeneity in MPF over distance than linear trend in MPF over distance (a non-significant gradient of -0**·**003 per km, 95%CI -0**·**006 to 0**·**0003, p=0**·**08), suggesting that geographical heterogeneity had a greater effect than any bias that may be introduced by distance from the hospital. Furthermore, we found that the incidence of malaria on active case detection from 2 cohorts monitored over 10 years [9, 10] correlated closely with MPF measured at the hospital (r=0**·**84, p<0**·**001, S3 Fig).” |
| Study size | 10 | Explain how the study size was arrived at  The sample size was all eligible children admitted during the 25 years of surveillance |
| Quantitative variables | 11 | Explain how quantitative variables were handled in the analyses. If applicable, describe which groupings were chosen and why  >*In the descriptive analysis age was categorized to two groups (<=1 year old and >1 year old) this was informed by the hypothesis that MPF for children <1year old would indicate transmission intensity without the offsetting effect of acquired immunity. This was described in the manuscript as;*  “In order to disentangle the effects of loss of immunity from transmission intensity, we hypothesized that MPF for children <1 year old (MPF_<1yr_) would indicate transmission intensity without the offsetting of acquired immunity (as has been done previously [11, 12]).”  >*In the logistic regression model age, community ITN use and EVI were used as continuous variables* |
| Statistical methods | 12 | (*a*) Describe all statistical methods, including those used to control for confounding  *>Statistical methods are describe in the Paper as follows;*  “A t-test or the Wilcoxon rank-sum test was used where appropriate to compare average/median age between time points. The spatio-temporal heterogeneity of malaria transmission in Kilifi County was assessed using a multivariable logistic regression model with the presence or absence of malaria parasites by microscopy among medical admissions (excluding trauma and elective surgery) as the outcome variable, time (continuous variable to capture the secular trends), year (categorical to capture year-to-year variability), region (referring to North vs South of the creek), location (referring to administrative areas within region) and space-time interactions as the independent covariates. Multiple Fractional Polynomials were used to assess nonlinear associations in regression models.” |
|  |  | (*b*) Describe any methods used to examine subgroups and interactions  >No subgroup analysis was conducted. Age-time interaction and space-time interactions ware included in the Multiple Fractional Polynomial model. |
|  |  | (*c*) Explain how missing data were addressed  *>Missing data was addressed and reported in the manuscript as follows;*  “Observations with missing locations, age and slide result were excluded prior to the analysis. Missing ITN data were included in the descriptive analysis but excluded from the logistic regression analysis. No data imputation was done at any stage in the analysis.” |
|  |  | (*d*) *Cohort study*—If applicable, explain how loss to follow-up was addressed  Not Applicable  *Case-control study*—If applicable, explain how matching of cases and controls was addressed  Not Applicable  *Cross-sectional study*—If applicable, describe analytical methods taking account of sampling strategy  Not Applicable |
|  |  | (*e*) Describe any sensitivity analyses  *>A sensitivity analysis based on the parasite threshold >2500 was performed on the data and reported in the manuscript as follows;*  “A sensitivity analysis was carried out with “malaria case” defined using a cut-off parasitaemia of >2,500 parasites per µl.” |

Continued on next page

| Results | | |
| --- | --- | --- |
| Participants | 13* | (a) Report numbers of individuals at each stage of study—eg numbers potentially eligible, examined for eligibility, confirmed eligible, included in the study, completing follow-up, and analysed  These has been reported in the manuscript as a flow chart (Fig 1) |
|  |  | (b) Give reasons for non-participation at each stage  Reasons have been highlighted Fig 1 |
|  |  | (c) Consider use of a flow diagram  Fig 1 |
| Descriptive data | 14* | (a) Give characteristics of study participants (eg demographic, clinical, social) and information on exposures and potential confounders  Has been described in the manuscript using a table of characteristics (Table 1 ) |
|  |  | (b) Indicate number of participants with missing data for each variable of interest  Missing data has been reported in the manuscript in (Table 1) we conducted Multiple imputations of ITN use and compared the result with Complete case analysis (S2 Table). Characteristics of individuals with missing ITN data during community surveys is reported in S3 Table |
|  |  | (c) *Cohort study*—Summarise follow-up time (eg, average and total amount) |
| Outcome data | 15* | *Cohort study*—Report numbers of outcome events or summary measures over time  Not Applicable |
|  |  | *Case-control study—*Report numbers in each exposure category, or summary measures of exposure  Numbers in each exposure category has been reported in the manuscript in (Table 1) |
|  |  | *Cross-sectional study—*Report numbers of outcome events or summary measures  Not Applicable |
| Main results | 16 | (*a*) Give unadjusted estimates and, if applicable, confounder-adjusted estimates and their precision (eg, 95% confidence interval). Make clear which confounders were adjusted for and why they were included  *These was reported in the manuscript as;*  “Personal ITN use was a predictor in univariable analysis (OR=0**·**73, 95%CI 0**·**65 to 0**·**83, p=<0**·**001), but not after adjusting for ITN use in the 2 km radius around the admitted children’s residences (S2 Table).” After adjusting for age. |
|  |  | (*b*) Report category boundaries when continuous variables were categorized  Not Applicable |
|  |  | (*c*) If relevant, consider translating estimates of relative risk into absolute risk for a meaningful time period  Not Applicable |
| Other analyses | 17 | Report other analyses done—eg analyses of subgroups and interactions, and sensitivity analyses  Presented in the Manuscript as follows;  “Similar results were obtained using a model that included clustering at the 250 square meters (S4 Table). We repeated our analysis using a cut-off of 2,500 parasites per µl. The same patterns were seen: i.e. a post-decline increase in MPF among older children (S6 Fig), and inverse relationship between MPF and age of malaria (r=-0**·**63, p<0**·**001), and of protection by community-level ITN use (S7 Fig). These patterns remained statistically significant.” |
| Discussion | | |
| Key results | 18 | Summarise key results with reference to study objectives  Presented in the Manuscript as follows;  “The proportion of acutely unwell children admitted to hospital with malaria positive slides (i.e. the MPF) declined from 2002 until 2009 (as reported previously [13]). However, after 2009 there was an increase in MPF that continued through to 2014. This increase was mostly seen in older children: there were relatively modest increases among children less than 1 year of age (MPF_<1yr_), but much greater increases among children above 1 year of age (MPF_>1yr_). This combination of findings suggests that the increase in malaria is due to increasing susceptibility to disease among older children, exacerbated by a slight increase in underlying transmission intensity since 2009. Additional evidence for increasing transmission after 2009 can be seen in the increasing prevalence of asymptomatic parasitaemia (PfPR) among children admitted for trauma, and work showing increasing parasite prevalence throughout the Kenyan Coast [14]. “ |
| Limitations | 19 | Discuss limitations of the study, taking into account sources of potential bias or imprecision. Discuss both direction and magnitude of any potential bias  > Presented in the Manuscript as follows;  “Study limitations include the use of MPF among acute admissions which may be biased by varying access to care. In support of the use of MPF, we found a close correlation between location-matched MPFs with the incidence rates from active case detection data from two cohort studies conducted within the same study area. Furthermore we showed that geographic heterogeneity was larger than any consistent bias in MPF with distance to the hospital (S2 Fig), agreeing with previous work [11].  >We based our analysis on the proportion of children with positive malaria slides. This case definition includes children with chronic asympomtomatic parasitaemia and coincident fever. To exclude a bias resulting from including these children we repeated the analysis after applying a threshold of >2,500 parasites per µl, which excludes the majority of children with asymptomatic parasitaemia [9] (S6 Fig).  > Our ITN surveys were conducted annually and based on reported rather than observed use. The potential misclassification could lead to an underestimate of the protective effect of ITNs.  > ITN use has not been randomly assigned and therefore confounding could be present. If present this would most likely dilute the effect size (i.e. ITN use would be better taken up in areas of higher malaria risk). Furthermore observational studies of ITN use have consistently shown similar findings to RCT suggesting that within specific communities confounding is not pronounced [15].” |
| Interpretation | 20 | Give a cautious overall interpretation of results considering objectives, limitations, multiplicity of analyses, results from similar studies, and other relevant evidence  Presented in the Manuscript as follows;  “We show that despite substantial reductions in malaria transmission in Kilifi, residual malaria transmission has continued and older children are increasingly vulnerable to disease. As countries and regions make progress in malaria control [16], maintaining control measures will be essential: in fact further progress will be required to offset the increasing rates of malaria in older children. Achieving ITN coverage close to 100% shows promise as a strategy, but will require novel strategies, particularly among groups that seem less keen to use ITNs such as adolescent males and young adults [17].” |
| Generalisability | 21 | Discuss the generalisability (external validity) of the study results  Presented in the Manuscript as follows;  “Our data were taken from a single geographical setting. However there is evidence that immunity to malaria is dependent on exposure in a wide range of geographical settings [2] and therefore our findings may be relevant more widely across Africa in settings where transmission is falling [18]. “ |
| Other information | | |
| Funding | 22 | Give the source of funding and the role of the funders for the present study and, if applicable, for the original study on which the present article is based  Presented in the Manuscript as follows;  “The work was supported by core funding from the Wellcome Trust. RWS, AMN, TNW are supported by the Wellcome Trust as Principal Research Fellow (#079080/#103602), Intermediate Fellow (#095127) and Senior Fellow (#091758), respectively, and PB is supported by the UK Medical Research Council (MRC) and the UK Department for International Development (DFID) under the MRC/DFID Concordat agreement” |

*Give information separately for cases and controls in case-control studies and, if applicable, for exposed and unexposed groups in cohort and cross-sectional studies.

**Note:** An Explanation and Elaboration article discusses each checklist item and gives methodological background and published examples of transparent reporting. The STROBE checklist is best used in conjunction with this article (freely available on the Web sites of PLoS Medicine at http://www.plosmedicine.org/, Annals of Internal Medicine at http://www.annals.org/, and Epidemiology at http://www.epidem.com/). Information on the STROBE Initiative is available at [www.strobe-statement.org](http://www.strobe-statement.org/).

**References**

1. Woolhouse ME. Patterns in parasite epidemiology: the peak shift. Parasitol Today. 1998;14(10):428-34. Epub 2006/10/17. PubMed PMID: 17040835.

2. Okiro EA, Al-Taiar A, Reyburn H, Idro R, Berkley JA, Snow RW. Age patterns of severe paediatric malaria and their relationship to Plasmodium falciparum transmission intensity. Malar J. 2009;8:4. Epub 2009/01/09. doi: 10.1186/1475-2875-8-4. PubMed PMID: 19128453; PubMed Central PMCID: PMCPmc2630996.

3. Karema C, Aregawi MW, Rukundo A, Kabayiza A, Mulindahabi M, Fall IS, et al. Trends in malaria cases, hospital admissions and deaths following scale-up of anti-malarial interventions, 2000-2010, Rwanda. Malar J. 2012;11:236. Epub 2012/07/25. doi: 10.1186/1475-2875-11-236. PubMed PMID: 22823945; PubMed Central PMCID: PMCPmc3502144.

4. Trape JF, Tall A, Diagne N, Ndiath O, Ly AB, Faye J, et al. Malaria morbidity and pyrethroid resistance after the introduction of insecticide-treated bednets and artemisinin-based combination therapies: a longitudinal study. Lancet Infect Dis. 2011;11(12):925-32. Epub 2011/08/23. doi: 10.1016/s1473-3099(11)70194-3. PubMed PMID: 21856232.

5. Pemberton-Ross P, Smith TA, Hodel EM, Kay K, Penny MA. Age-shifting in malaria incidence as a result of induced immunological deficit: a simulation study. Malar J. 2015;14:287. Epub 2015/07/25. doi: 10.1186/s12936-015-0805-1. PubMed PMID: 26206255; PubMed Central PMCID: PMCPmc4513612.

6. Scott JA, Bauni E, Moisi JC, Ojal J, Gatakaa H, Nyundo C, et al. Profile: The Kilifi Health and Demographic Surveillance System (KHDSS). Int J Epidemiol. 2012;41(3):650-7. Epub 2012/05/01. doi: 10.1093/ije/dys062. PubMed PMID: 22544844; PubMed Central PMCID: PMCPmc3396317.

7. Midekisa A, Senay G, Henebry GM, Semuniguse P, Wimberly MC. Remote sensing-based time series models for malaria early warning in the highlands of Ethiopia. Malar J. 2012;11:165. Epub 2012/05/16. doi: 10.1186/1475-2875-11-165. PubMed PMID: 22583705; PubMed Central PMCID: PMCPmc3493314.

8. Tatem AJ, Goetz SJ, Hay SI. Terra and Aqua: new data for epidemiology and public health. Int J Appl Earth Obs Geoinf. 2004;6(1):33-46. Epub 2004/11/01. PubMed PMID: 22545030; PubMed Central PMCID: PMCPmc3337546.

9. Bejon P, Berkley JA, Mwangi T, Ogada E, Mwangi I, Maitland K, et al. Defining childhood severe falciparum malaria for intervention studies. PLoS Med. 2007;4(8):e251. Epub 2007/08/24. doi: 10.1371/journal.pmed.0040251. PubMed PMID: 17713980; PubMed Central PMCID: PMCPmc1949845.

10. Mwangi TW, Ross A, Snow RW, Marsh K. Case definitions of clinical malaria under different transmission conditions in Kilifi District, Kenya. J Infect Dis. 2005;191(11):1932-9. PubMed PMID: 15871128.

11. Bejon P, Williams TN, Nyundo C, Hay SI, Benz D, Gething PW, et al. A micro-epidemiological analysis of febrile malaria in Coastal Kenya showing hotspots within hotspots. Elife. 2014;3:e02130. Epub 2014/05/21. doi: 10.7554/eLife.02130. PubMed PMID: 24843017; PubMed Central PMCID: PMCPmc3999589.

12. Snow RW, Molyneux C, Warn P, Omumbo J, Nevill C, Gupta S, et al. Infant parasite rates and immunoglobulin M seroprevalence as a measure of exposure to Plasmodium falciparum during a randomized controlled trial of insecticide-treated bed nets on the Kenyan coast. Am J Trop Med Hyg. 1996;55(2):144-9.

13. O'Meara WP, Bejon P, Mwangi TW, Okiro EA, Peshu N, Snow RW, et al. Effect of a fall in malaria transmission on morbidity and mortality in Kilifi, Kenya. Lancet. 2008;372(9649):1555-62. Epub 2008/11/06. doi: 10.1016/s0140-6736(08)61655-4. PubMed PMID: 18984188; PubMed Central PMCID: PMCPmc2607008.

14. Snow RW, Kibuchi E, Karuri SW, Sang G, Gitonga CW, Mwandawiro C, et al. Changing Malaria Prevalence on the Kenyan Coast since 1974: Climate, Drugs and Vector Control. PLoS One. 2015;10(6):e0128792. Epub 2015/06/25. doi: 10.1371/journal.pone.0128792. PubMed PMID: 26107772.

15. Lim SS, Fullman N, Stokes A, Ravishankar N, Masiye F, Murray CJ, et al. Net benefits: a multicountry analysis of observational data examining associations between insecticide-treated mosquito nets and health outcomes. PLoS Med. 2011;8(9):e1001091. Epub 2011/09/13. doi: 10.1371/journal.pmed.1001091. PubMed PMID: 21909249; PubMed Central PMCID: PMCPmc3167799.

16. Noor AM, Kinyoki DK, Mundia CW, Kabaria CW, Mutua JW, Alegana VA, et al. The changing risk of Plasmodium falciparum malaria infection in Africa: 2000-10: a spatial and temporal analysis of transmission intensity. Lancet. 2014;383(9930):1739-47. Epub 2014/02/25. doi: 10.1016/s0140-6736(13)62566-0. PubMed PMID: 24559537; PubMed Central PMCID: PMCPmc4030588.

17. Noor AM, Kirui VC, Brooker SJ, Snow RW. The use of insecticide treated nets by age: implications for universal coverage in Africa. BMC Public Health. 2009;9:369. Epub 2009/10/03. doi: 1471-2458-9-369 [pii]

10.1186/1471-2458-9-369. PubMed PMID: 19796380; PubMed Central PMCID: PMC2761895.

18. O'Meara WP, Mangeni JN, Steketee R, Greenwood B. Changes in the burden of malaria in sub-Saharan Africa. The Lancet Infectious Diseases. 2010;10(8):545-55.
